# Synthesis of Okaramine M, Its Conversion to Amauromines, and Concise Bidirectional and Biomimetic Total Synthesis of Amauromines

**DOI:** 10.1021/acs.orglett.5c02088

**Published:** 2025-06-29

**Authors:** Daniel Schmelzer, Christian B. W. Stark

**Affiliations:** Department of Chemistry, Institute for Organic Chemistry, 14915 University of Hamburg, Martin-Luther-King-Platz 6, D-20146 Hamburg, Germany

## Abstract

A synthesis of *exo*- and *endo*-okaramine M and their conversion into different sets of stereoisomers of amauromines are reported. Moreover, a short bidirectional and biomimetic total synthesis of amauromines is described using an Ir-catalyzed double prenylation as the key step. Accordingly, amauromine with its six stereogenic centers can be prepared in two synthetic steps in enantiomerically pure form starting from unprotected l-tryptophan.

## Graphical abstract

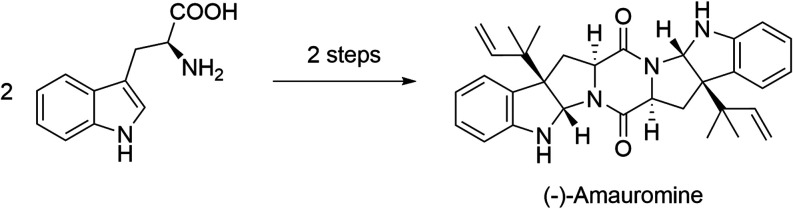

The amauromines are a small family of highly symmetric indole alkaloids belonging to the larger group of hexahydropyrrolo indole natural products.^1^ The main representatives of the amauromines are the three diastereomers amauromine **1a**, epiamauromine **1b**, and novoamauromine **1c** (Figure 1).

**Figure 1:**
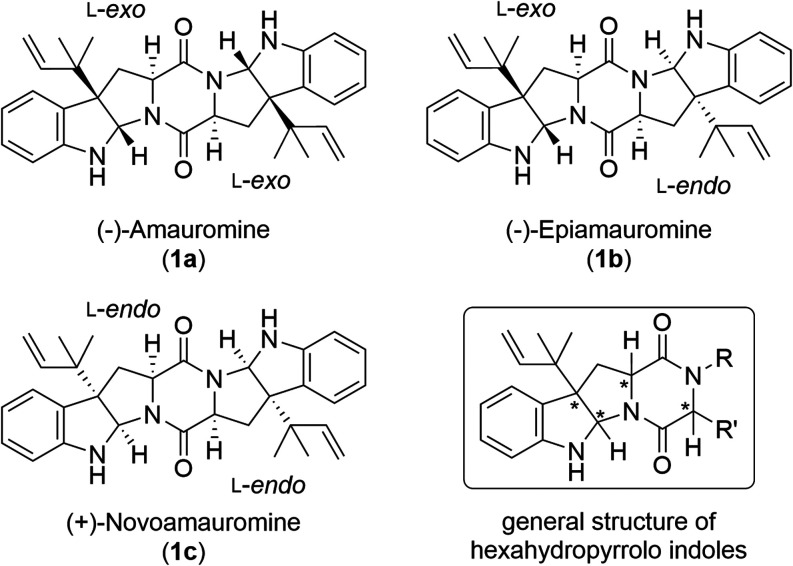
Structures of the three naturally occurring diastereomeric amauromines **1a**, **1b**, and **1c** and general core structure of reverse prenylated hexahydropyrrolo indole natural products.

The amauromines have been isolated from different fungal sources. Amauromine itself (**1a**) was originally found to be produced by *Amauroascus* sp. and was the first member of the group of reverse prenylated hexahydropyrrolo indole natural products whose absolute configuration was determined.^2^ In an almost simultaneous report, the constitution of a compound named nigrifortine, isolated from *Penicillium nigricans*, was published, but neither its relative nor absolute configuration was established.^3^ From the published spectroscopic data, it can however be concluded that nigrifortine is actually identical with amauromine (**1a**). Epiamauromine (**1b**) is produced by *Aspergillus ochraceus*,^4^ while novoamauromine (**1c**), the third member of the family, was isolated from *Aspergillus novofumigatus*.^5^ None of the fungal producers seem to form mixtures of the three diastereomeric amauromines but rather generate each isomer fully stereoselectively (*vide infra*).

Early publications report that amauromine (**1a**) exhibits potent vasodilating activity.^2^ Other members of the amauromines have been found to display moderate insecticidal^4^ and anticancer activity.^5^ More interestingly, it has recently been shown by Müller and König et al. that amauromine (**1a**) acts as a selective cannabinoid receptor antagonist (CB_1_) at nanomolar levels.^6^ It has therefore been suggested as a starting point for the development of a new chemical class of therapeutic agents. A rapid synthetic access to amauromines^7^ (and derivatives thereof) is therefore highly desirable.

Based on the chemoenzymatic syntheses and investigations of the biosynthesis of amauromines^8^ and related hexahydropyrrolo indole natural products,^9,10^ it is likely that l-tryptophan-derived diketopiperazine dimer **2** (Scheme 1) is the early key intermediate in the biosynthesis of all amauromines. Interestingly, the same dimer (**2**) seems to be a biosynthetic intermediate of all okaramines (Scheme 1).^11^ Due to its central role as a key intermediate in both biosynthetic pathways, we suggest that this natural product be designated (which has not been named previously) as *pre*-okamauromine (Scheme 1). Despite the different and diverse prenylation patterns of okaramines as compared to amauromines, okaramine M can be regarded as another bridging link between the two biosynthetic pathways. Accordingly, prenylation of the common congener *pre*-okamauromine (**2**) by means of an electrophilic addition of a prenyl cation equivalent to the C3 position of one of the two identical indole heterocycles would lead to an iminium ion intermediate (**3** in Scheme 1), which in turn is trapped by the proximal nitrogen nucleophile of the central diketopiperazine.^8–10^ Subsequent N-acetylation in *Penicillium simplicissimum* (ATCC 90288) yields okaramine M (R = Ac (**4a**)). Even if to date no such monoprenylated intermediates have been isolated from any of the amauromine producers, it has to be assumed that it in fact is an intermediate in the biosynthesis of amauromines, as well.^8–10^ Thus, a second prenylation of the still unreacted indole heterocycle (of **5a**) with the same intramolecular trapping of the iminium ion intermediate would directly lead to the amauromines. With respect to the stereochemical course of the reaction(s), the second prenylation can occur either from the same face as the initial prenylation, yielding *C*_2_-symmetric amauromine (**1a**) or novoamauromine (**1c** (not shown in Scheme 1)) or from opposite sides, leading to epiamauromine (**1b**). It is worth noting that in the latter case two prenyltransferases acting with antipodal facial selectivity are required to produce the observed configuration of the natural product.

**Scheme 1:**
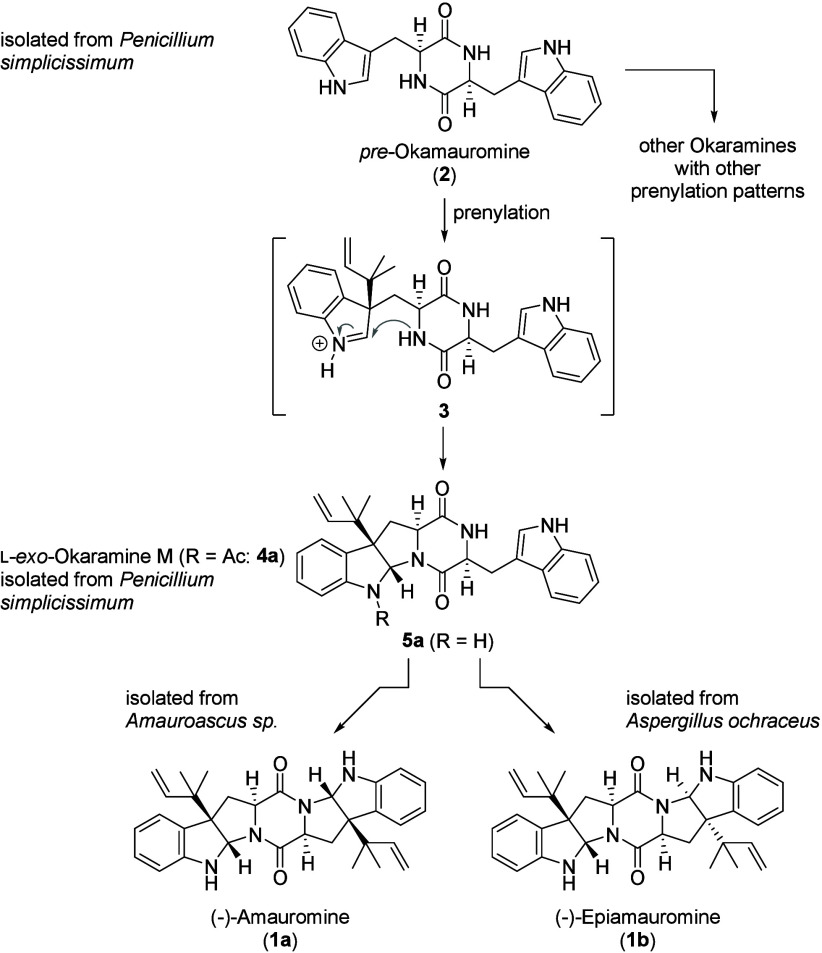
Proposed Biosynthesis of l-*exo*-Okaramine M and Amauromines from *pre*-Okamauromine

We here report a synthesis of different stereoisomers of okaramine M^12^ and investigations into their conversion into amauromines as well as stereochemical implications thereof. In addition, a facile and concise bidirectional direct route to amauromines is described.

Our synthesis followed the biosynthetic pathway (Scheme 1) and commenced with the preparation of the known symmetrical l-tryptophan-based diketopiperazine **2** (*pre*-okamauromine) anticipated to be the early key intermediate in the biosynthesis of okaramines and all three amauromines.^8–11^ Different approaches to this initial target compound have been reported previously.^13^ In our hands, the best results were achieved using appropriately protected tryptophans.^13c^ Thus, equimolar amounts of *N*-Boc-protected l-tryptophan **6** were condensed with l-tryptophan methyl ester **7** using HOBt and EDC in the presence of triethyl amine (Scheme 2). After standard Boc deprotection using TFA- and hydroxylamine-mediated cyclocondensation, diketopiperazine **2** was isolated in a 44% yield over three steps (Scheme 2). Direct condensation of the free amino acid (**9**) in ethylene glycol at 180 °C was less effective and afforded the desired *pre*-okamauromine (**2**) in only 10% yield, albeit using only a single-step transformation and a more readily available starting material (unprotected l-tryptophan **9** itself (Scheme 2)).^13d^

**Scheme 2:**
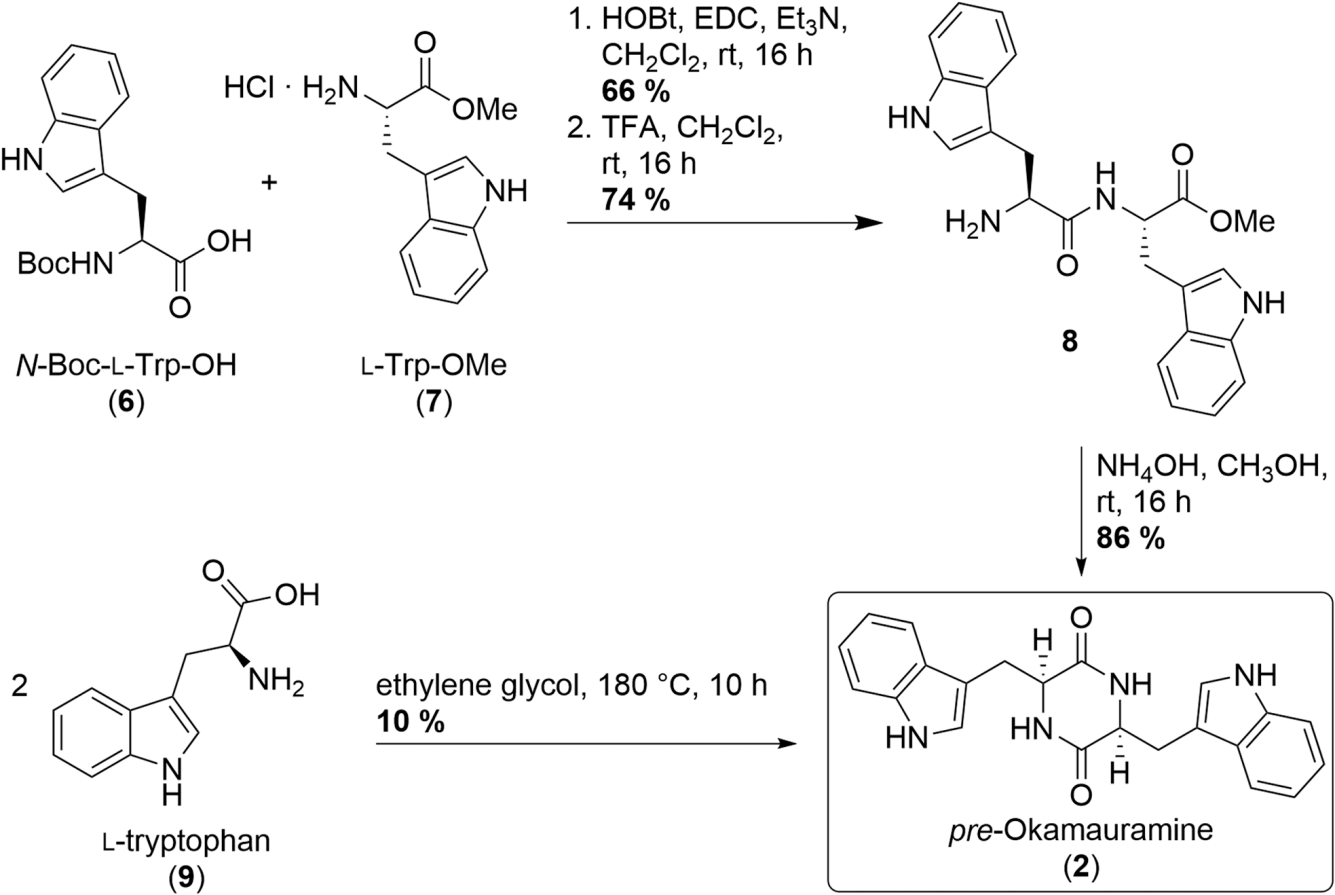
Two Routes to *pre*-Okamauromine

In order to access okaramine M (**4**), we decided to monoprotect this *C*_2_-symmetric intermediate at one of the indole nitrogens (Scheme 3). TIPS-protected *pre*-okamauromine **10** was then subjected to Ir-catalyzed prenylation^14,15^ in the presence of triethyl borane and achiral phosphoramidite ligand **L1**.^15^ These nonasymmetric conditions were chosen in order to access both the originally reported structure of okaramine M (l-*endo*-**4b**)^11^ and the revised stereoisomer (l-*exo*-**4a**).^12a^ In addition, both diastereoisomers are actually required in order to access all three natural amauromines (*vide infra*). The prenylation delivered the desired product in a yield of 89% with full regioselectivity in favor of the branched isomer. The two possible diastereoisomers l-*exo*-**11a** and l-*endo*-**11b** were formed with negligible selectivity of 1.5:1 reflecting a low degree of substrate control.^7f,14^ A slight preference in favor of the thermodynamic (cf. Scheme 4) *exo*-product (**11a**) was detected. The two stereoisomers could be separated by standard flash chromatography. Starting from l-*exo*-**11a**, N-acetylation followed by TBAF (buffered with stoichiometric amounts of acetic acid)-mediated TIPS deprotection gave natural l-*exo*-okaramine M (**4a**) in a yield of 71% over two steps.^12a^ When the same two-step process was applied to the minor diastereoisomer l-*endo*-**11b**, the product with the originally published configuration (l-*endo*-okaramine M (**4b**)) was isolated in a yield of 64% over two steps (Scheme 3).^11^

**Scheme 3:**
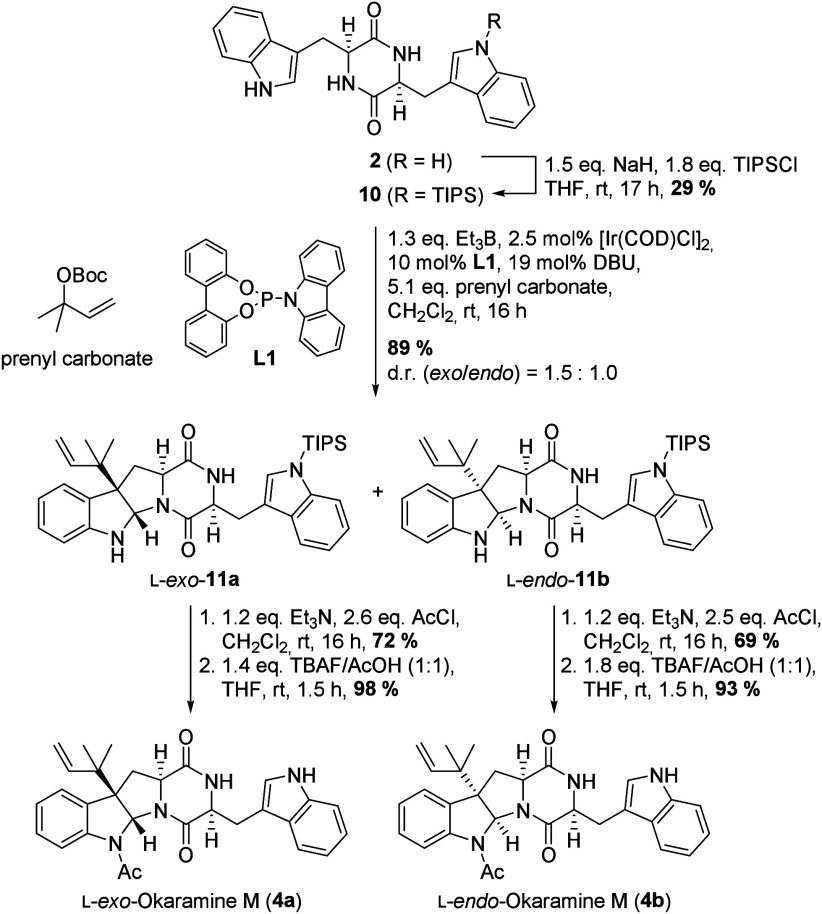
Synthesis of l-*exo*- and l-*endo*-Okaramine M

**Scheme 4:**
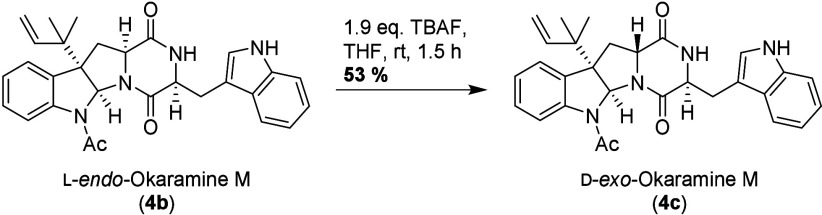
Synthesis of d-*exo*-Okaramine M

Another unnatural isomer of okaramine M can be readily accessed by simply stirring l-*endo*-okaramine M (**4b**) in the presence of TBAF in THF. Under the slightly basic reaction regime, full epimerization to d-*exo*-okaramine M (**4c**) is achieved (Scheme 4). Considering the existence of prenyltransferases with antipodal facial selectivity (*vide supra*),^8–10^ it seems not unlikely that l-*endo*-okaramine M (**4b**) and d-*exo*-okaramine M (**4c**) may actually be natural products, as well, but have thus far not been isolated from natural sources.^16,17^ In this respect, it is worth noting that the biosynthesis of novoamauromine (**1c**) indeed requires the intermediary formation of deacyl-l-*endo*-okaramine M **5b** (*vide infra*).

We next investigated the conversion of l-*exo*- and l-*endo*-deacyl-okaramine M into amauromines (**1**). To this end, the silyl protecting group of both diastereoisomers was removed to restore the indole C3 nucleophilicity, and then both diastereoisomers were independently subjected to the prenylation procedure using the standard Ir-*pre*-catalyst ([Ir­(COD)­Cl]_2_) in the presence of triethyl borane and achiral phosphoramidite ligand **L1** (Scheme 5, top half).^15^ While allylic alkylation of l-*exo*-isomer **5a** gave a mixture of amauromine **1a** and epiamauromine **1b** in 88% yield and a ratio of 1.4:1.0, the same reaction using l-*endo*-**5b** as the starting material produced a mixture of epiamauromine **1b** and novoamauromine **1c**. Thus, the two diastereomeric natural products were obtained in a combined yield of 84% and in a ratio of 2:1 (**1b**:**1c**).

**Scheme 5:**
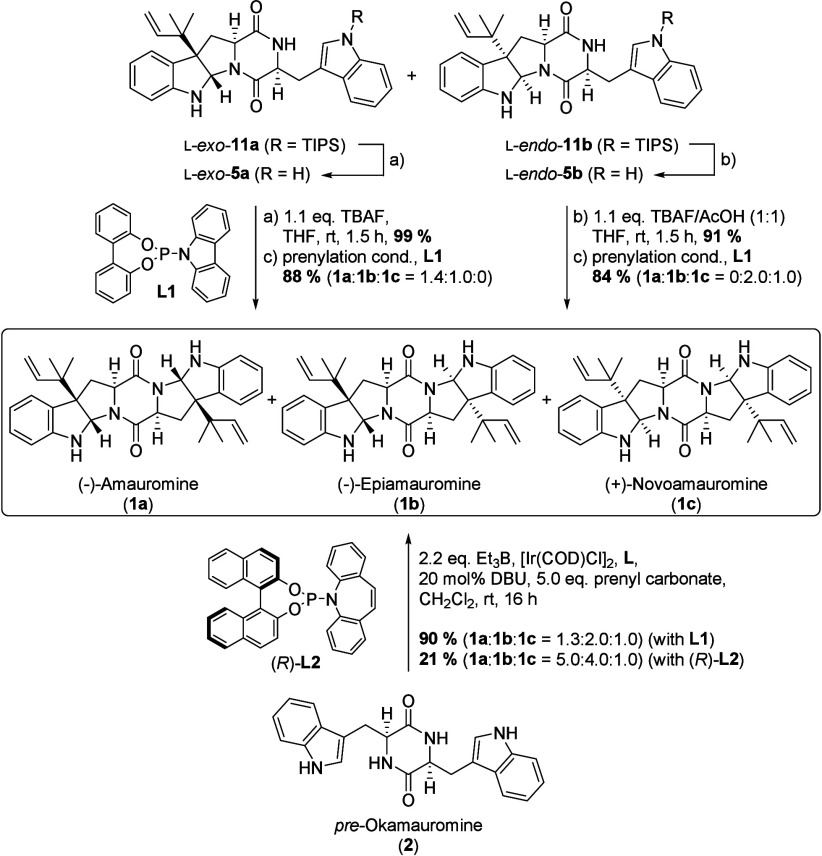
Biomimetic Conversion of Deacyl-Okaramines M into Different Stereoisomers of Amauromine and Single-Step Conversion of *pre*-Okamauromine M to Amauromines

Finally, direct access to amauromines by means of a (putatively biomimetic^18^) double prenylation of *pre*-okamauromine (**2**), rather than the stepwise prenylation procedure via deacyl-okaramine M, was explored. We therefore subjected *pre*-okamauromine **2** directly to the standard prenylation conditions using the standard Ir-*pre*-catalyst in the presence of triethyl borane and achiral phosphoramidite ligand **L1** (Scheme 5, bottom half).^15^ To our delight, no mixtures of regioisomers or products with varying degrees of alkylation were formed, but a clean and direct conversion to amauromines was observed. Again, the reaction proceeded with extraordinarily high regioselectivity with respect to both the nucleophile and the electrophile. Thus, the three amauromines were isolated in a combined yield of 90% (Scheme 5, bottom half). As expected from the experiments for the conversion of okaramines M to amauromines (Scheme 5, top half), no significant diastereoselectivity was observed but rather an almost statistical distribution of the three possible diastereoisomers (**1a**:**1b**:**1c** = 1.3:2.0:1.0). Again, a slight preference for the thermodynamic *exo*-*exo*-product (amauromine **1a**) was determined (Scheme 5, bottom half). We next investigated a set of different asymmetric prenylation procedures^14,15^ in order to test whether either amauromine (**1a**) (or novoamauromine (**1c**)) could be prepared with a reasonable degree of selectivity. Among a range of different chiral ligands and asymmetric prenylation protocols,^14,15^ the application of Carreira’s procedure in the presence of chiral phosphoramidite ligand (*R*)-**L2** gave the best results with respect to facial selectivity, resulting in a product ratio of 5.0:4.0:1.0 (Scheme 5, bottom half). However, it must be noted that the combination of the bulky substrates and the sterically highly congested Ir catalysts with their chiral ligands led to extremely slow reactions, low conversions, and consequently low isolated yields (rarely surpassing 50%).

In summary, we have presented a short and biomimetic synthesis of different stereoisomers of the reverse prenylated indole alkaloid okaramine M (**4**) starting from common *C*_2_-symmetric precursor *pre*-okamauromine (**2**). l-*exo*- and l-*endo*-okaramine M (**4a** and **4b**, respectively) were then converted into different sets of stereoisomers of amauromines. Finally, a direct and putatively also biomimetic conversion of *pre*-okamauromine to amauromines was achieved. The reaction gave an excellent isolated yield of 90%. Formation of two new C–C bonds and two new C–N bonds occurred with excellent regioselectivity, producing the nanomolar CB_1_ inhibitor amauromine (**1**) in only two synthetic operations starting from l-tryptophan. No protecting groups are required, and the product with its six stereogenic centers is isolated in enantiomerically pure form. Initial investigations toward the asymmetric control of the double prenylation to produce one of the amauromines preferentially were carried out.

## Supplementary Material



## Data Availability

The data underlying this study are available in the published article and its Supporting Information.

## Notes

Experimental procedures, characterization data, and reproduction of NMR spectra (PDF)

## References

[1] a Williams, R. M. ; Stocking, E. M. ; Sanz-Cervera, J. F. Biosynthesis of Prenylated Alkaloids Derived from Tryptophan. In Biosynthesis; Leeper, F. J. , Vederas, J. C. , Eds.; Topics in Current Chemistry, Vol. 209; Springer, 2000; pp 97–173.

[2] Takase S., Kawai Y., Uchida I., Tanaka H., Aoki H. (1984). Structure of Amauromine, A New Alkaloid with Vasodilating Activity
Produced by Amauroascus Sp. Tetrahedron Lett..

[3] Laws I., Mantle P. G. (1985). Nigrifortine, a
Diketopiperazine Metabolite of Penicillium
Nigricans. Phytochemistry.

[4] de
Guzman F. S., Gloer J. B., Wicklow D. T., Dowd P. F. (1992). New Diketopiperazine
Metabolites from the Sclerotia of Aspergillus Ochraceus. J. Nat. Prod..

[5] Ishikawa K., Hosoe T., Itabashi T., Wakana D., Takizawa K., Yaguchi T., Kawai K.-I. (2010). Novoamauromine and *ent*-Cycloechinulin: Two New Diketopiperazine Derivatives from Aspergillus
novofumigatus. Chem. Pharm. Bull..

[6] Elsebai M. F., Rempel V., Schnakenburg G., Kehraus S., Müller C. E., König G. M. (2011). Identification
of a Potent and Selective Cannabinoid CB1 Receptor Antagonist from
Auxarthron reticulatum. ACS Med. Chem. Lett..

[7] Takase S., Itoh Y., Uchida I., Tanaka H., Aoki H. (1985). Total Synthesis of Amauromine. Tetrahedron
Lett..

[8] Yin W.-B., Yu X., Xie X.-L., Li S.-M. (2010). Preparation of pyrrolo­[2,3-*b*]­indoles carrying a
β-configured reverse C3-dimethylallyl
moiety by using a recombinant prenyltransferase CdpC3PT. Org. Biomol. Chem..

[9] Yin W.-B., Grundmann A., Cheng J., Li S.-M. (2009). Acetylaszonalenin biosynthesis in
Neosartorya fischeri. Identification of the biosynthetic gene cluster
by genomic mining and functional proof of the genes by biochemical
investigation. J. Biol. Chem..

[10] Li S.-M. (2009). Applications
of dimethylallyltryptophan synthases and other indole prenyltransferases
for structural modification of natural products. Appl. Microbiol. Biotechnol..

[11] Shiono Y., Akiyama K., Hayashi H. (1999). New Okaramine
Congeners, Okaramine
J, K, L, M and Related Compounds, from Penicillium simplicissimum
ATCC 90288. Biosci. Biotechnol. Biochem..

[12] Iizuka T., Takiguchi S., Kumakura Y., Tsukioka N., Higuchi K., Kawasaki T. (2010). First total
synthesis and stereochemical revision of okaramine M. Tetrahedron Lett..

[13] b Walker, K. L. ; Loach, R. P. ; Movassaghi, M. Total Synthesis of Complex 2,5-Diketopiperazine Alkaloids. In The Alkaloids: Chemistry and Biology; Knölker, H.-J. , Ed.; Academic Press: Cambridge, U.K., 2023; Vol. 90, pp 159–206. 10.1016/bs.alkal.2023.06.002 PMC10955524 37716796

[14] Qu J., Helmchen G. (2017). Applications of Iridium-Catalyzed
Asymmetric Allylic Substitution Reactions in Target-Oriented Synthesis. Acc. Chem. Res..

[15] Ruchti J., Carreira E. M. (2014). Ir-Catalyzed Reverse
Prenylation of 3-Substituted Indoles: Total Synthesis of (+)-Aszonalenin
and (−)-Brevicompanine B. J. Am. Chem.
Soc..

[16] Hetzler B. E., Trauner D., Lawrence A. L. (2022). Natural
product anticipation through
synthesis. Nat. Rev. Chem..

[17] Schmidt J., Khalil Z., Capon R. J., Stark C. B. W. (2014). Heronapyrrole
D: A case of co-inspiration of natural product biosynthesis, total
synthesis and biodiscovery. Beilstein J. Org.
Chem..

[18] Li X.-W., Nay B. (2014). Transition metal-promoted
biomimetic steps in total syntheses. Nat. Prod.
Rep..

