# Supplementary material for: Synthesis of Okaramine M, Its Conversion to Amauromines, and Concise Bidirectional and Biomimetic Total Synthesis of Amauromines
Source: Org Lett. 2025 Jun 29;27(27):7367–71. doi: 10.1021/acs.orglett.5c02088 (PMC12261399; doi:10.1021/acs.orglett.5c02088)
Supplement: Supplementary file 1 [file ol5c02088_si_001.pdf]

Supporting Information

Synthesis of Okaramine M, Its Conversion to Amauromines, and Concise Bidirectional and Biomimetic Total Synthesis of Amauromines

Daniel Schmelzer and Christian B. W. Stark*

Department of Organic Chemistry, University of Hamburg, Martin-Luther-King-Platz 6, 20146 Hamburg, Germany

Table of contents

1 General experimental conditions SI-1

2 Synthetic Procedures and Characterization Data SI-1

2.1 Synthesis of *pre*-Okamauromine (**2**) SI-1

2.2 Synthesis of (3*S*,6*S*)-3-((1*H*-Indol-3-yl)methyl)-6-((1-(triisopropylsilyl)-1*H*-indol-3-yl)methyl)piperazine-2,5-dione (**10**) SI-3

2.3 Synthesis of (3*S*,5a*S*,10b*R*,11a*S*)-10b-(2-Methylbut-3-en-2-yl)-3-((1-(triisopropylsilyl)-1*H*-indol-3-yl)methyl)-2,3,6,10b,11,11a-hexahydro-4*H*-pyrazino[1',2':1,5]pyrrolo[2,3-*b*]indole-1,4(5a*H*)-dione (L-*exo*-**11a**) and (3*S*,5a*R*,10b*S*,11a*S*)-10b-(2-Methylbut-3-en-2-yl)-3-((1-(triisopropylsilyl)-1*H*-indol-3-yl)methyl)-2,3,6,10b,11,11a-hexahydro-4*H*-pyrazino-[1',2':1,5]pyrrolo[2,3-*b*]indole-1,4(5a*H*)-dione (L-*endo*-**11b**) SI-5

2.4 Synthesis of (3*S*,5a*R*,10b*R*,11a*S*)-6-Acetyl-10b-(2-methylbut-3-en-2-yl)-3-((1-(triisopropylsilyl)-1*H*-indol-3-yl)methyl)-2,3,6,10b,11,11a-hexahydro-4*H*-pyrazino[1',2':1,5]pyrrolo[2,3-*b*]indole-1,4(5a*H*)-dione (*N*-acyl-L-*exo*-**11a**) SI-9

2.5 Synthesis of (3*S*,5a*S*,10b*S*,11a*S*)-6-Acetyl-10b-(2-methylbut-3-en-2-yl)-3-((1-(triisopropylsilyl)-1*H*-indol-3-yl)methyl)-2,3,6,10b,11,11a-hexahydro-4*H*-pyrazino[1',2':1,5]pyrrolo[2,3-*b*]indole-1,4(5a*H*)-dione (*N*-acyl-L-*endo*-**11b**) SI-11

2.6 Synthesis of (3*S*,5a*S*,10b*R*,11a*S*)-3-((1*H*-Indol-3-yl)methyl)-10b-(2-methyl-but-3-en-2-yl)-2,3,6,10b,11,11a-hexahydro-4*H*-pyrazino[1',2':1,5]pyrrolo-[2,3-*b*]indole-1,4(5a*H*)-dione (L-*exo*-**5a**) SI-13

2.7 Synthesis of (3*S*,5a*R*,10b*S*,11a*S*)-3-((1*H*-Indol-3-yl)methyl)-10b-(2-methyl-but-3-en-2-yl)-2,3,6,10b,11,11a-hexahydro-4*H*-pyrazino[1',2':1,5]pyrrolo-[2,3-*b*]indole-1,4(5a*H*)-dione (L-*endo*-**5b**) SI-15

2.8 Synthesis of L-*exo*-Okaramine M (**4a**) SI-17

2.9 Synthesis of L-*endo*-Okaramine M (**4b**) SI-19

2.10 Synthesis of D-*exo*-Okaramine M (**4c**) SI-21

2.11 Synthesis of Amauromine (**1a**), Epiamauromine (**1b**) and Novoamauromine (**1c**) SI-23

3 References SI-29

1 General experimental conditions

General Techniques. All reagents were used as purchased from commercial suppliers. Solvents were purified by conventional methods prior to use. Reactions were monitored by **thin layer chromatography** using Machery-Nagel pre-coated TLC-sheets ALUGRAM® Xtra SIL G/UV254 and visualized with ninhydrin [0.8 g ninhydrin, 200 mL ethanol], potassium permanganate [(2.4 g $KMnO_4$, 16 g K_2CO_3, 4 mL NaOH (5 %), 196 mL H_2O)] or ceric ammonium molybdate [(phosphomolybdic acid (5 g), $Ce(SO_4)_2 \cdot 2\ H_2O$ (2 g), H_2SO_4 conc. (12 mL), H_2O (188 mL)]. **Chromatographic purification** was performed as flash chromatography on Fluka silica gel 60 (particle size 0.040-0.063 mm). Yields refer to chromatographically purified and spectroscopically pure compounds. **NMR spectra** were recorded on a Bruker AV-300 (operating at 300 MHz for ^1H and 76 MHz for ^{13}C acquisitions), Bruker AV-400 (operating at 400 MHz for ^1H and 101 MHz for ^{13}C acquisitions), a Bruker AV-500 (operating at 500 MHz for ^1H and 126 MHz for ^{13}C acquisitions) or a Bruker AV-600 (operating at 600 MHz for ^1H and 151 MHz for ^{13}C acquisitions). Chemical shifts δ are reported in ppm with the solvent resonance as the internal standard: chloroform-*d* ($CDCl_3$): 7.26 (^1H-NMR), 77.16 (^{13}C-NMR); methanol-*d*4 (CD_3OD): 3.31 (^1H-NMR), 49.00 (^{13}C-NMR); acetone-*d*6: 2.05 (^1H-NMR), 206.26 (^{13}C-NMR). Coupling constants *J* are given in Hertz (Hz). Multiplicities are classified as follows: s = singlet, d = doublet, t = triplet, q = quartet, qui = quintet, sept = septet and combinations thereof, or m = multiplet or br = broad signal. Two-dimensional NMR (H,H-COSY, HSQC, HMBC, NOESY) were used for the assignment of all resonance signals. The relative stereochemistry of all synthesized compounds, especially *exo*- and *endo*-configurations, was assigned with the help of NOESY-NMR and comparison of literature data. For simplicity, the numbering of the carbon atoms of a given structure does not necessarily follow IUPAC rules. The chosen numbering system for each compound can be found in the SI. **High resolution mass spectra** were obtained on an Agilent 6224 ESI-TOF. **IR spectra** were recorded on a Bruker ALPHA FT-IR Platinum ATR. Wave numbers $\tilde{\nu}$ are reported in reciprocal centimeters (cm^{-1}). **Optical rotation data** were measured with a Krüss Optronic P8000 at 598 nm using a 100 mm path-length cell in the solvent, at the concentration and temperature indicated. **Melting Points** were measured with a Büchi Melting Point M-565 and are uncorrected. All compounds were named according to IUPAC rules.

2 Synthetic Procedures and Characterization Data

2.1 Synthesis of *pre*-Okamauromine (2)

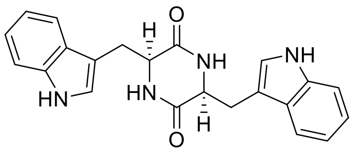

2

$C_{22}H_{20}N_4O_2$

372.43 g/mol

The synthesis of **2** was performed as described by Kieffer *et al.*[1] and resulted in a total yield of 44 % (37 mg, 0.099 mmol) over three steps. The analytical data of **2** obtained *via* that procedure is shown below.

Alternatively, the synthesis was performed directly from L-tryptophan as described by Jubeen *et al.*[2] but resulted in a yield of only 10 %, different to what was reported in the literature.

R$_f$: 0.38 (CH_2Cl_2/CH_3OH = 10:1). **Mp.**: 224-225 °C. **$[\alpha]_D^{20}$** = - 147.6 ° (*c* = 0.91, CH_3OH). **^{1}H-NMR** (600 MHz, CD_3OD): δ = 7.46 (dt, ^3J = 8.0 Hz, ^4J = 1.0 Hz, 2H), 7.31 (dt, ^3J = 8.2 Hz, ^4J = 0.9 Hz, 2H), 7.10 (ddd, ^3J = 8.2 Hz, ^3J = 7.0 Hz, ^4J = 1.2 Hz, 2H), 7.01 (ddd, ^3J = 8.0 Hz, ^3J = 7.0 Hz, ^4J = 1.0 Hz, 2H), 6.47 (s, 2H), 4.05 (ddd, ^3J = 7.4 Hz, ^3J = 4.0 Hz, ^5J = 0.9 Hz, 2H), 2.93 (ddd, ^2J = 14.4 Hz, ^3J = 3.9 Hz, ^5J = 0.8 Hz, 2H), 2.18 (dd, ^2J = 14.4 Hz, ^3J = 7.3 Hz, 2H) ppm. **^{13}C-NMR** (151 MHz, CD_3OD): δ = 169.7, 138.1, 128.6, 125.9, 122.5, 120.1, 119.7, 112.4, 109.5, 56.9, 31.4 ppm. **FT-IR** (ATR): $\tilde{\nu}$ = 3396, 3314, 3048, 2913, 1660, 1455, 1324, 1091, 737, 422 cm^{-1}. **HR-MS** (ESI) *m/z*: $[M+H]^+$ calc. for $C_{22}H_{21}N_4O_2^+$ = 373.1659, found = 373.1632.

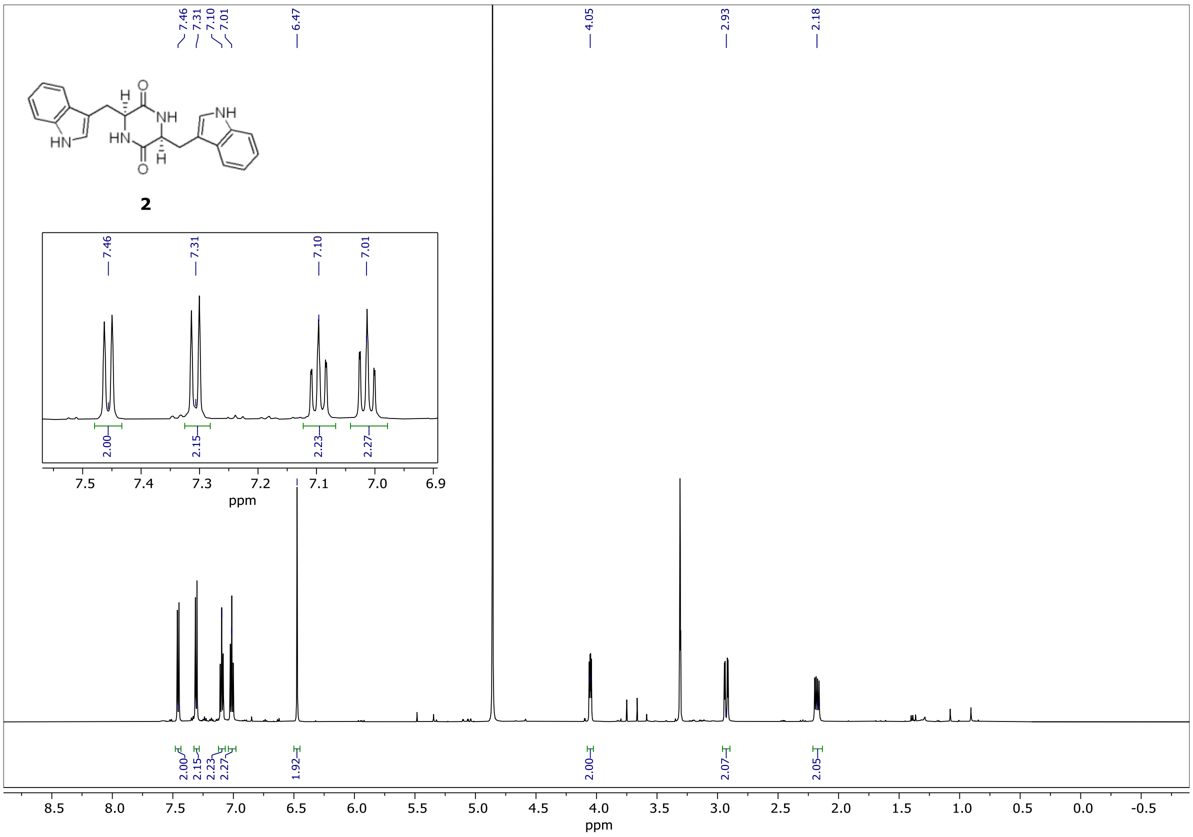

Spectrum 1. ^{1}H-NMR (600 MHz, CD_3OD) of **2**.

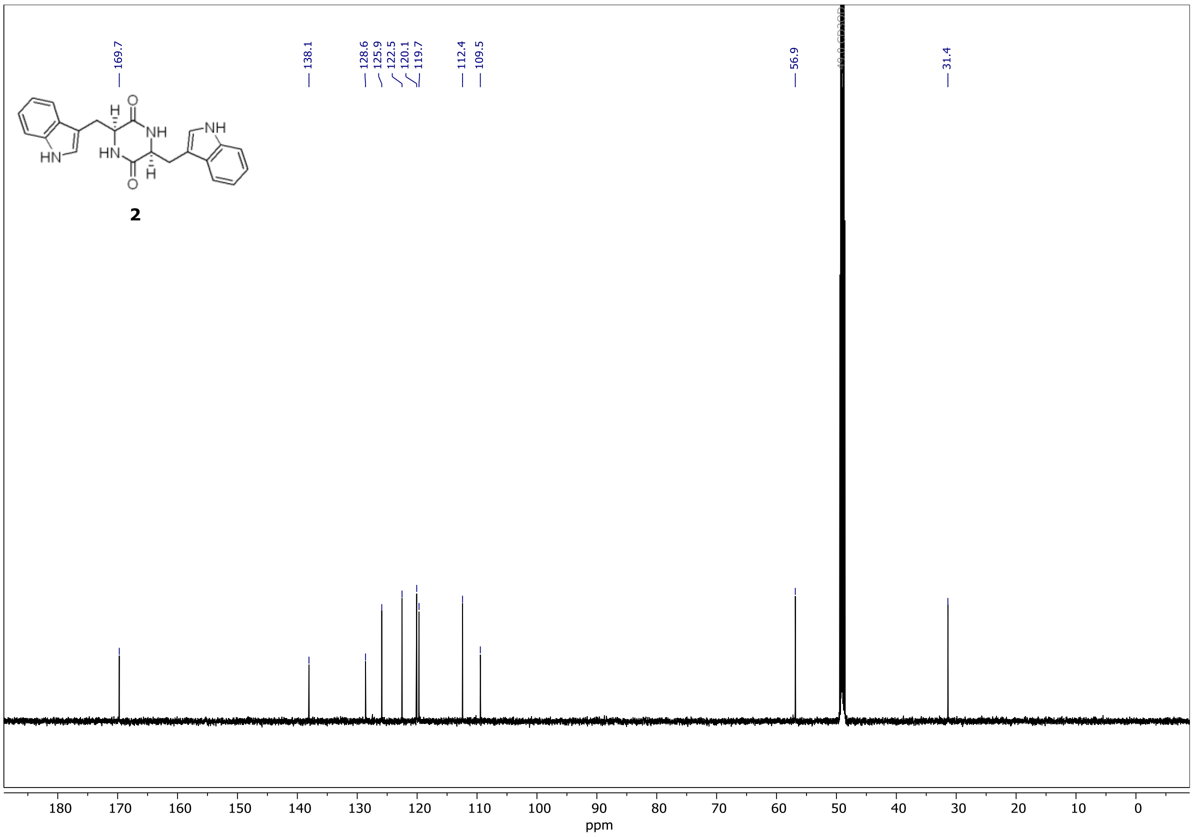

Spectrum 2. ^{13}C-NMR (151 MHz, CD_3OD) of **2**.

2.2 Synthesis of (3*S*,6*S*)-3-((1*H*-Indol-3-yl)methyl)-6-((1-(triisopropylsilyl)-1*H*-indol-3-yl)methyl)-piperazine-2,5-dione (10)

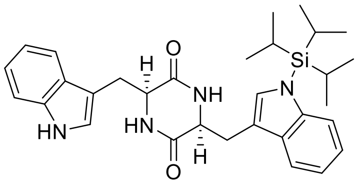

10

$C_{31}H_{40}N_4O_2Si$

528.77 g/mol

pre-Okamauromine (**2**) (485.5 mg, 1.3 mmol, 1.0 eq.) was suspended in abs. THF (18 mL). Sodium hydride (77.2 mg, 1.9 mmol, 1.5 eq.) was added portionwise and the reaction mixture stirred for 1.5 h. After that, triisopropylsilyl chloride (0.50 mL, 0.46 g, 2.4 mmol, 1.8 eq.) was added and the solution was stirred for additional 17 h. Distilled water (20 mL) was added, the mixture was extracted three times with ethyl acetate and the combined organic layers were dried over $MgSO_4$. After removing the solvent *in vacuo*, the crude product was purified *via* column chromatography (CH_2Cl_2/CH_3OH = 20:1). *N*-TIPS-*pre*-Okamauromine (**10**) (201.6 mg, 0.381 mmol, 29 %) was obtained as a white solid.

R_f: 0.34 (EtOAc). **Mp.**: 243-244 °C. $[\alpha]_D^{20}$ = - 49.3 ° (*c* = 1.0, $CHCl_3$). **^{1}H-NMR** (500 MHz, $CDCl_3$): δ = 8.13 (s, 1H), 7.58 – 7.49 (m, 2H), 7.47 (d, ^3J = 8.0 Hz, 1H), 7.34 (d, ^3J = 8.1 Hz, 1H), 7.24 – 7.08 (m, 4H), 6.76 (s, 1H),

6.41 (d, ^3J = 2.0 Hz, 1H), 5.96 (d, ^3J = 2.0 Hz, 1H), 5.82 (d, ^3J = 1.7 Hz, 1H), 4.21 – 4.15 (m, 2H), 3.25 – 3.17 (m, 2H), 2.55 (dd, ^2J = 14.5 Hz, ^3J = 7.9 Hz, 1H), 2.37 (dd, ^2J = 14.3 Hz, ^3J = 8.6 Hz, 1H), 1.66 (sept, ^3J = 7.5 Hz, 3H), 1.13 (d, ^3J = 7.5 Hz, 9H), 1.10 (d, ^3J = 7.5 Hz, 9H) ppm. **^{13}C-NMR** (126 MHz, $CDCl_3$): δ = 167.4, 167.1, 141.6, 136.4, 131.2, 130.6, 126.9, 124.4, 122.6, 122.1, 120.2, 120.0, 119.1, 119.0, 114.4, 111.4, 111.3, 109.2, 55.5, 55.2, 30.9, 30.7, 18.3, 18.2, 12.9 ppm. **FT-IR** (ATR): $\tilde{\nu}$ = 3414, 2947, 2868, 1671, 1453, 1324, 1138, 961, 882, 739, 687, 650, 582, 519, 448, 425 cm^{-1}. **HR-MS** (ESI) *m*/*z*: $[M+H]^+$ calc. for $C_{31}H_{41}N_4O_2Si^+$ = 529.2993, found = 529.2969.

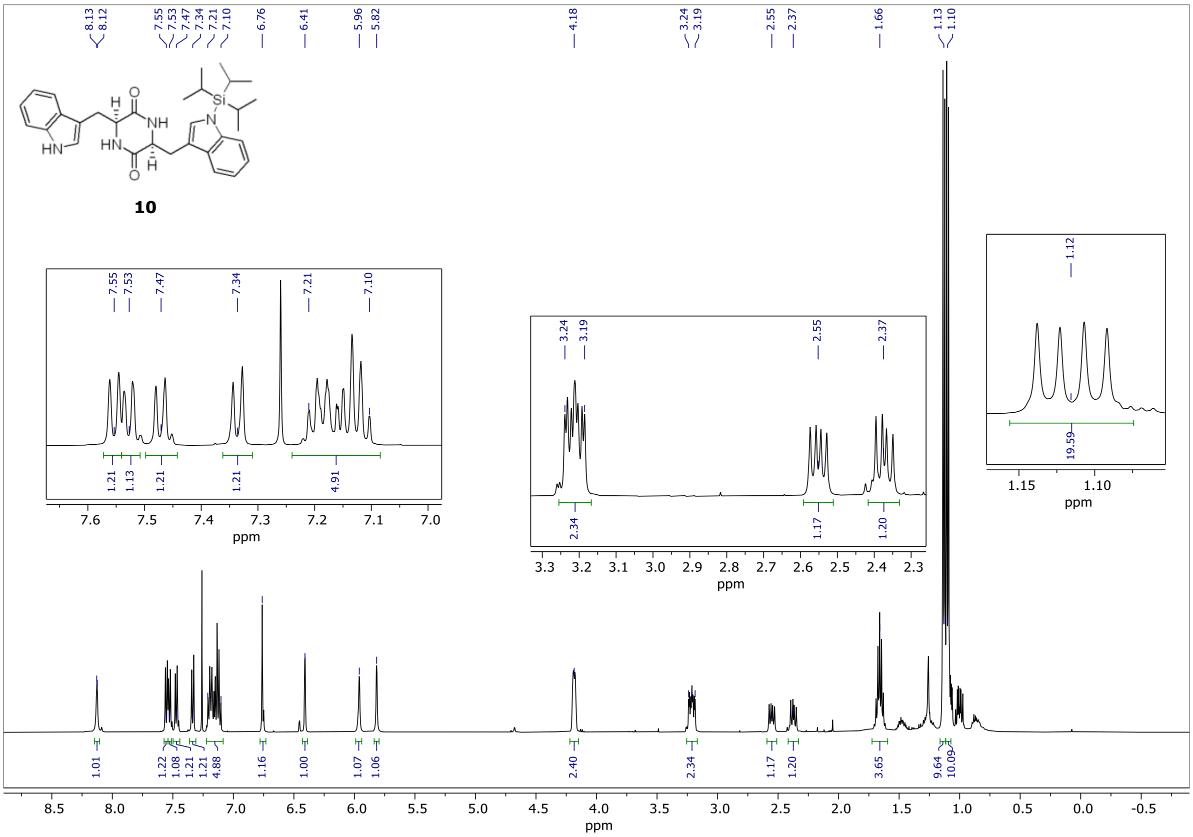

Spectrum 3. ^{1}H-NMR (500 MHz, $CDCl_3$) of **10**.

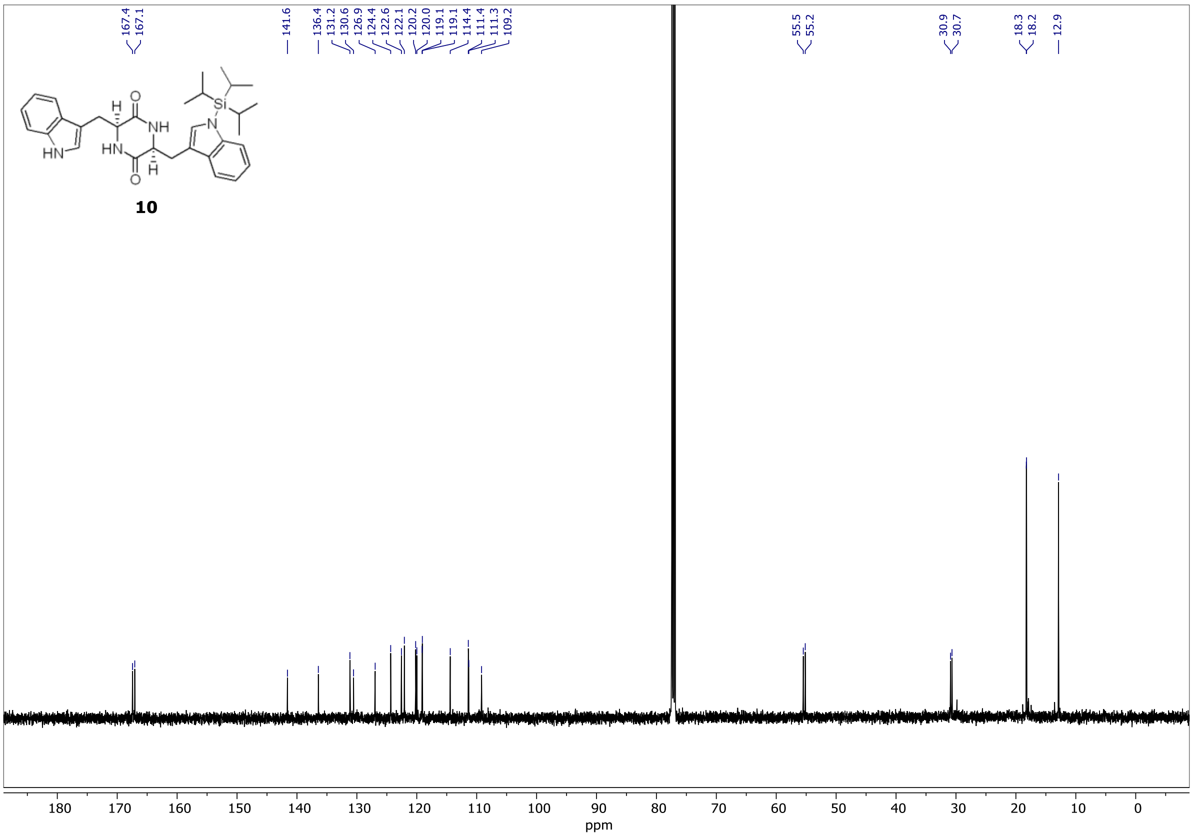

Spectrum 4. ^{13}C-NMR (126 MHz, $CDCl_3$) of **10**.

2.3 Synthesis of (3*S*,5a*S*,10b*R*,11a*S*)-10b-(2-Methylbut-3-en-2-yl)-3-((1-(triisopropylsilyl)-1*H*-indol-3-yl)methyl)-2,3,6,10b,11,11a-hexahydro-4*H*-pyrazino[1',2':1,5]pyrrolo[2,3-*b*]indole-1,4(5a*H*)-dione (L-*exo*-11a) and (3*S*,5a*R*,10b*S*,11a*S*)-10b-(2-Methylbut-3-en-2-yl)-3-((1-(triisopropylsilyl)-1*H*-indol-3-yl)methyl)-2,3,6,10b,11,11a-hexahydro-4*H*-pyrazino[1',2':1,5]pyrrolo[2,3-*b*]indole-1,4(5a*H*)-dione (L-*endo*-11b)

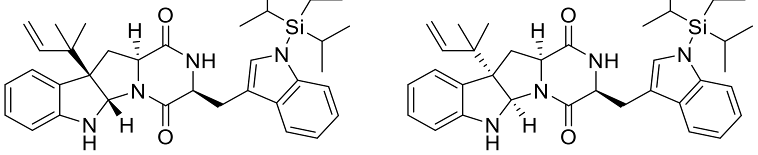

L-*exo*-**11a** L-*endo*-**11b**

$C_{36}H_{48}N_4O_2Si$

596.89 g/mol

The reaction was performed under inert conditions.

N-TIPS-*pre*-Okamauromine (**10**) (200 mg, 0.378 mmol, 1.0 eq.) was dissolved in dichloromethane (4.5 mL), triethyl borane (0.50 mL, 0.50 mmol, 1.3 eq., 1.0 M in hexane) was added and the solution was stirred for 10 min. $[Ir(COD)Cl]_2$ (6.3 mg, 9.5 µmol, 2.5 mol%) and **L1** (15.2 mg, 40.0 µmol, 11 mol%) were dissolved in dichloromethane (0.75 mL) and stirred for 10 min upon which a bright red solution was obtained. DBU (11 µL, 11 mg, 72 µmol, 19 mol%) was added to the substrate solution and the catalyst solution was added directly

afterwards. *tert*-Butyl-(2-methylbut-3-en-2-yl) carbonate (prenyl carbonate) (355 mg, 1.91 mmol, 5.1 eq.) was added and the reaction mixture was stirred for 20 h. After removing the solvent *in vacuo*, the crude product was purified *via* column chromatography (PE/EtOAc = 2:1).

Two isomers L-*exo*-**11a** and L-*endo*-**11b** (202 mg, 0.338 mmol, 89 %, dr = 1.5:1.0) could be separated during column chromatography and were obtained as white solids.

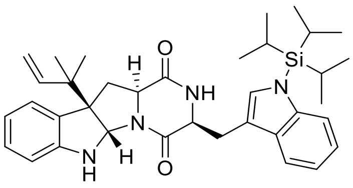

L-*exo*-**11a**

R*f*: 0.20 (PE/EtOAc = 2:1). **Mp.**: 120-121 °C. $[\alpha]_D^{20}$ = - 223.0 ° (*c* = 1.0, $CHCl_3$). **^{1}H-NMR** (600 MHz, $CDCl_3$): δ = 7.53 (dt, ^3J = 7.8 Hz, ^4J = 1.1 Hz, 1H), 7.50 (dd, ^3J = 8.2 Hz, ^4J = 1.0 Hz, 1H), 7.20 – 7.06 (m, 5H), 6.75 (dt, ^3J = 7.5 Hz, ^4J = 1.1 Hz, 1H), 6.60 (dd, ^3J = 7.9 Hz, ^4J = 1.0 Hz, 1H), 5.97 (dd, $^3J_{trans}$ = 17.4 Hz, $^3J_{cis}$ = 10.9 Hz, 1H), 5.60 (s, 1H), 5.56 (s, 1H), 5.12 (dd, $^3J_{cis}$ = 10.8 Hz, ^4J = 1.2 Hz, 1H), 5.08 (dd, $^3J_{trans}$ = 17.4 Hz, ^4J = 1.2 Hz, 1H), 5.04 (s, 1H), 4.31 (ddd, ^3J = 10.9 Hz, ^3J = 3.7 Hz, ^4J = 1.9 Hz, 1H), 3.92 (ddd, ^3J = 11.1 Hz, ^3J = 6.2 Hz, ^4J = 1.8 Hz, 1H), 3.72 (ddd, ^2J = 15.1 Hz, ^3J = 3.7 Hz, ^4J = 1.1 Hz, 1H), 2.98 (dd, ^2J = 15.1 Hz, ^3J = 10.9 Hz, 1H), 2.52 (dd, ^2J = 12.7 Hz, ^3J = 6.3 Hz, 1H), 2.43 (dd, ^2J = 12.7 Hz, ^3J = 11.1 Hz, 1H), 1.67 (sept, ^3J = 7.5 Hz, 3H), 1.14 (d, ^3J = 7.5 Hz, 9H), 1.13 (d, ^3J = 7.5 Hz, 9H), 1.11 (s, 3H), 1.01 (s, 3H) ppm. **^{13}C-NMR** (151 MHz, $CDCl_3$): δ = 169.0, 166.2, 150.1, 143.7, 141.8, 130.3, 130.2, 129.2, 129.1, 125.2, 122.4, 120.3, 119.0, 118.4, 114.6, 114.5, 111.7, 109.4, 77.9, 61.8, 59.2, 55.0, 41.0, 36.1, 27.1, 23.0, 22.6, 18.3, 18.2, 13.0 ppm. **FT-IR** (ATR): $\tilde{\nu}$ = 3368, 2947, 2868, 1676, 1450, 1308, 1213, 1145, 910, 734, 689, 649, 519 cm^{-1}. **HR-MS** (ESI) *m*/*z*: $[M+H]^+$ calc. for $C_{36}H_{49}N_4O_2Si^+$ = 597.3619, found = 597.3604.

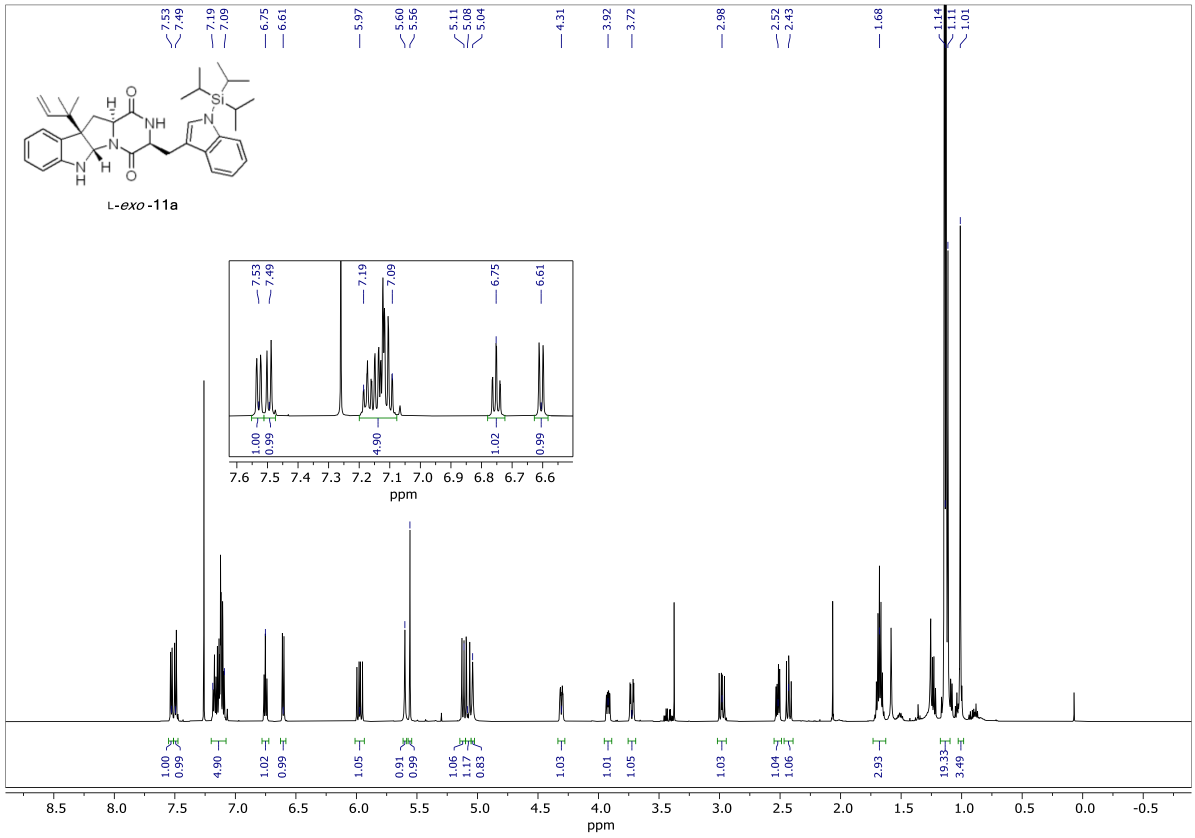

Spectrum 5. ^{1}H-NMR (600 MHz, $CDCl_3$) of L-*exo*-**11a**.

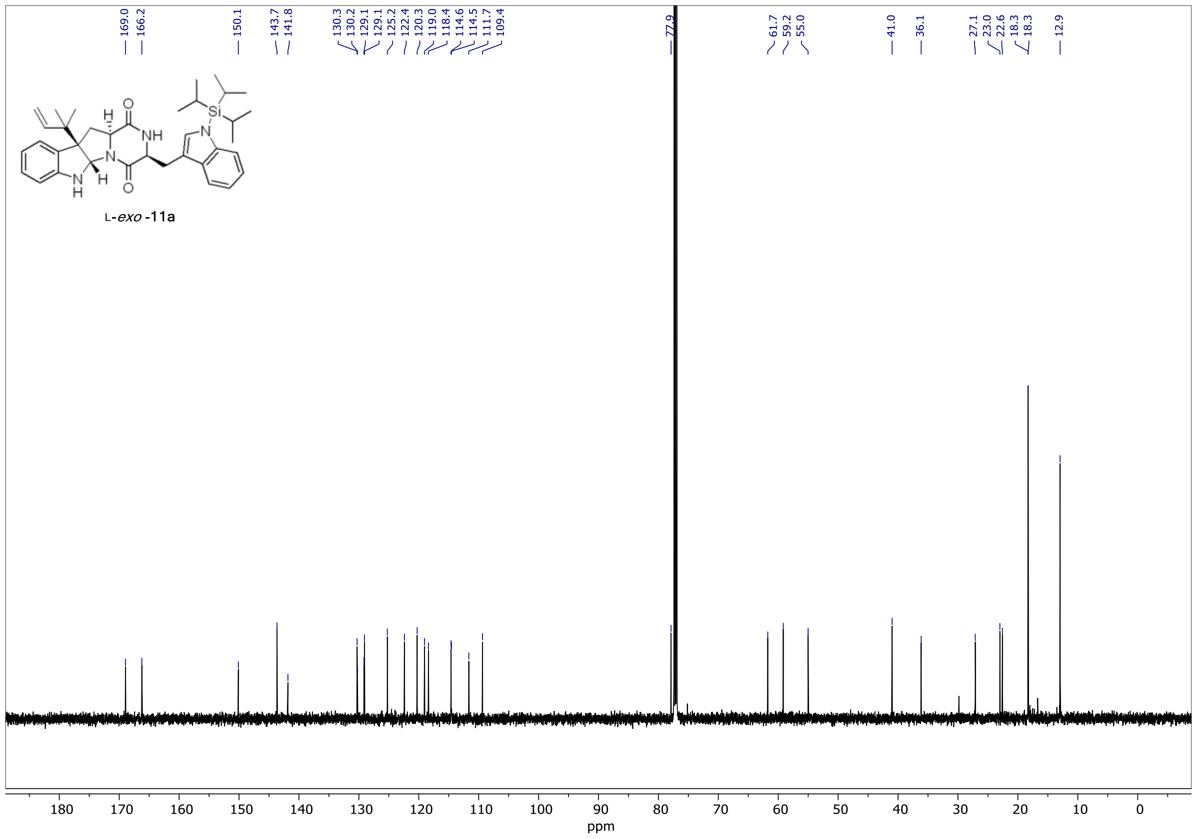

Spectrum 6. ^{13}C-NMR (151 MHz, $CDCl_3$) of L-*exo*-**11a**.

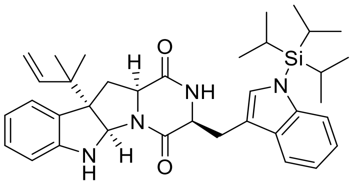

L-*endo*-**11b**

R*f*: 0.30 (PE/EtOAc = 2:1). **Mp.**: 114-115 °C. $[\alpha]_{\mathbf{D}}^{\mathbf{20}}$ = + 45.7 ° (*c* = 1.0, $CHCl_3$). **^{1}H-NMR** (600 MHz, $CDCl_3$): δ = 7.54 (dd, ^3J = 7.8 Hz, ^4J = 1.3 Hz, 1H), 7.49 (d, ^3J = 8.2 Hz, 1H), 7.21 – 7.07 (m, 4H), 7.09 (s, 1H), 6.74 (dt, ^3J = 7.5 Hz, ^4J = 1.1 Hz, 1H), 6.59 (d, ^3J = 7.8 Hz, 1H), 5.95 (dd, $^3J_{trans}$ = 17.4 Hz, $^3J_{cis}$ = 10.8 Hz, 1H), 5.51 – 5.48 (m, 1H), 5.43 (s, 1H), 5.16 (dd, $^3J_{cis}$ = 10.8 Hz, ^5J = 1.1 Hz, 1H), 5.13 (dd, $^3J_{trans}$ = 17.3 Hz, ^5J = 1.1 Hz, 1H), 4.32 – 4.28 (m, 1H), 4.09 (t, ^3J = 9.0 Hz, 1H), 3.72 (dd, ^2J = 15.0 Hz, ^3J = 3.6 Hz, 1H), 2.88 (dd, ^2J = 15.0 Hz, ^4J = 10.9 Hz, 1H), 2.80 (dd, ^2J = 13.8 Hz, ^3J = 8.9 Hz, 1H), 2.47 (dd, ^2J = 13.9 Hz, ^3J = 9.1 Hz, 1H), 1.65 (sept, ^3J = 7.5 Hz, 3H), 1.15 (s, 3H), 1.12 (d, ^3J = 7.3 Hz, 9H), 1.11 (d, ^3J = 7.3 Hz, 9H), 1.00 (s, 3H) ppm. **^{13}C-NMR** (151 MHz, $CDCl_3$): δ = 168.8, 168.2, 148.6, 143.7, 141.9, 131.7, 130.3, 130.1, 130.0, 128.5, 125.8, 122.4, 120.2, 118.9, 118.4, 114.8, 114.6, 111.7, 109.1, 79.7, 61.9, 58.0, 55.2, 41.8, 36.5, 27.1, 22.6, 22.5, 18.3, 18.2, 12.9 ppm. **FT-IR** (ATR): $\tilde{\nu}$ = 3375, 2948, 2867, 1667, 1451, 1414, 1309, 1130, 1016, 910, 883, 736, 689, 650, 518 cm^{-1}. **HR-MS** (ESI) *m/z*: $[M+H]^+$ calc. for $C_{36}H_{49}N_4O_2Si^+$ = 597.3619, found = 597.3612.

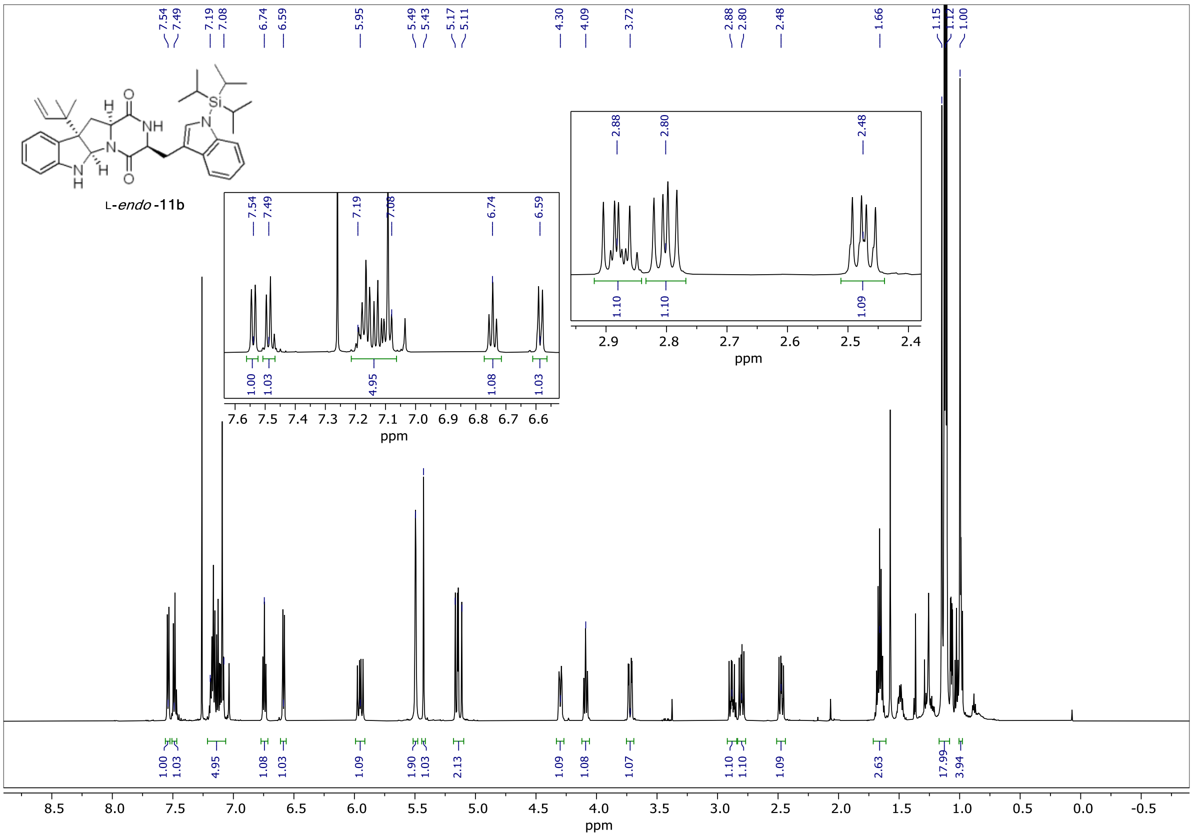

Spectrum 7. ^{1}H-NMR (600 MHz, $CDCl_3$) of L-*endo*-**11b**.

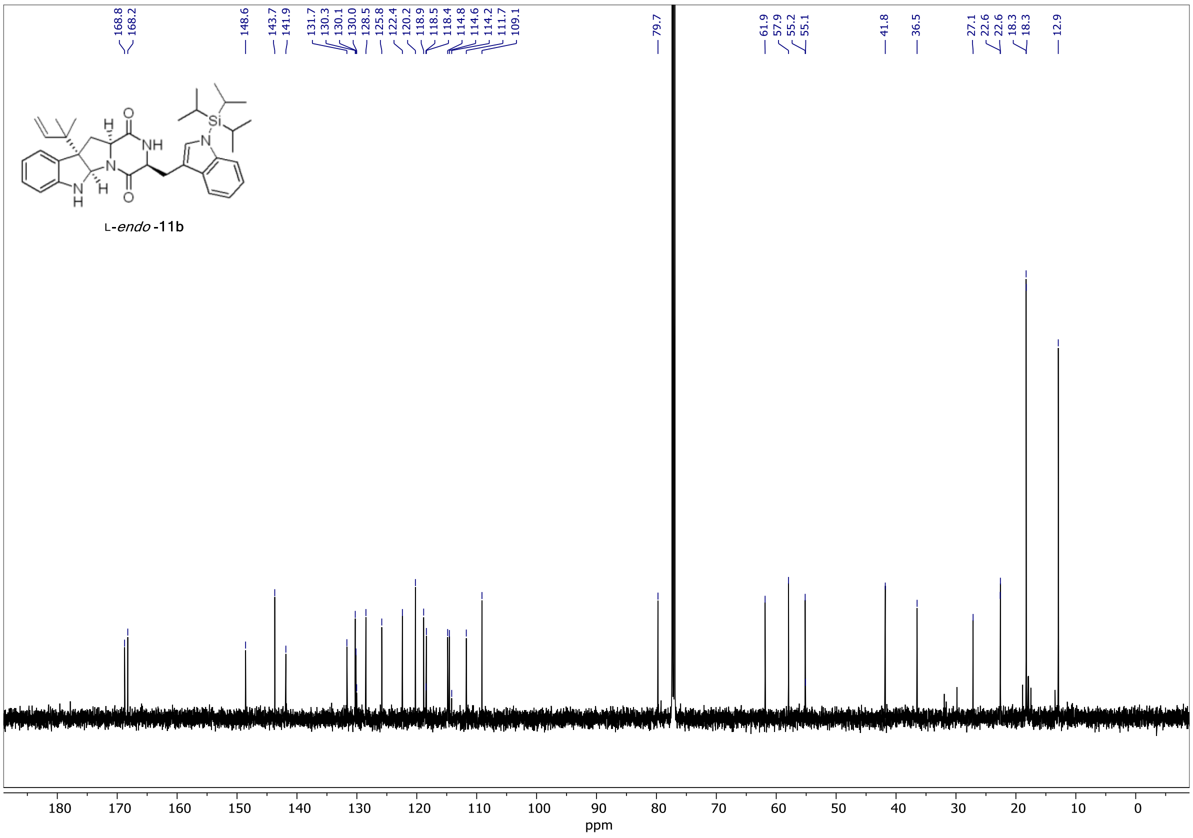

Spectrum 8. ^{13}C-NMR (151 MHz, $CDCl_3$) of L-*endo*-**11b**.

2.4 Synthesis of (3*S*,5a*R*,10b*R*,11a*S*)-6-Acetyl-10b-(2-methylbut-3-en-2-yl)-3-((1-(triisopropylsilyl)-1*H*-indol-3-yl)methyl)-2,3,6,10b,11,11a-hexahydro-4*H*-pyrazino[1',2':1,5]pyrrolo[2,3-*b*]indole-1,4(5a*H*)-dione (*N*-acyl-L-*exo*-11a)

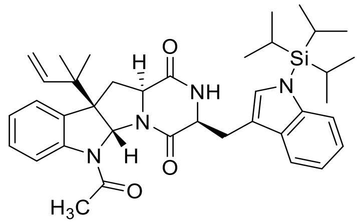

N-acyl-L-*exo*-**11a**

$C_{38}H_{50}N_4O_3Si$

638.93 g/mol

L-*exo*-**11a** (39.0 mg, 65.0 µmol, 1.0 eq.) was dissolved in dichloromethane (1 mL), triethylamine (11 µL, 7.9 mg, 78 µmol, 1.2 eq.) and acetyl chloride (12 µL, 13 mg, 0.17 mmol, 2.6 eq.) were added and the reaction mixture was stirred for 16 h. Then, more triethylamine (50 µL, 37 mg, 0.37 mmol, 5.7 eq.) was added and the solvent was removed *in vacuo*. The crude product was purified *via* column chromatography (PE/EtOAc = 1:1). *N*-acyl-L-*exo*-**11a** (34.2 mg, 47.0 µmol, 72 %) was obtained as a white solid.

R_f: 0.48 (PE/EtOAc = 1:2). **Mp.**: 121-122 °C. $[\alpha]_D^{20}$ = - 87.0 ° (c = 1.0, $CHCl_3$). **^{1}H-NMR** (300 MHz, $CDCl_3$): δ = 8.01 (s, 1H), 7.58 – 7.43 (m, 2H), 7.36 – 7.22 (m, 2H), 7.22 – 7.01 (m, 4H), 6.05 (s, 1H), 5.77 (dd, $^3J_{trans}$ = 17.1 Hz,

$^{3}J_{cis}$ = 11.2 Hz, 1H), 5.70 (s, 1H), 5.16 – 5.04 (m, 2H), 4.31 (dd, ^{3}J = 10.8 Hz, ^{3}J = 3.6 Hz, 1H), 3.80 (dd, ^{3}J = 11.5 Hz, ^{3}J = 5.6 Hz, 1H), 3.67 (dd, ^{2}J = 15.0 Hz, ^{3}J = 3.7 Hz, 1H), 3.07 – 2.91 (m, 1H), 2.69 (s, 3H), 2.57 (dd, ^{2}J = 12.5 Hz, ^{3}J = 5.7 Hz, 1H), 2.30 (t, ^{3}J = 12.0 Hz, 1H), 1.68 (sept, ^{3}J = 7.6 Hz, 3H), 1.21 – 1.05 (m, 21H), 0.96 (s, 3H) ppm. **^{13}C-NMR** (75 MHz, $CDCl_3$): δ = 170.3, 168.1, 165.6, 143.5, 143.2, 141.8, 130.3, 130.2, 129.2, 124.7, 122.4, 120.3, 119.4, 118.4, 114.7, 114.5, 111.4, 79.6, 61.1, 59.4, 54.9, 40.5, 36.1, 27.2, 23.8, 23.3, 22.4, 18.3, 12.9 ppm. **FT-IR** (ATR): $\tilde{\nu}$ = 2946, 2867, 1673, 1476, 1384, 1306, 1140, 1130, 1015, 963, 921, 882, 740, 688, 650, 566, 518, 427 cm^{-1}. **HR-MS** (ESI) *m/z*: $[M+H]^+$ calc. for $C_{38}H_{51}N_4O_3Si^+$ = 639.3725, found = 639.3728.

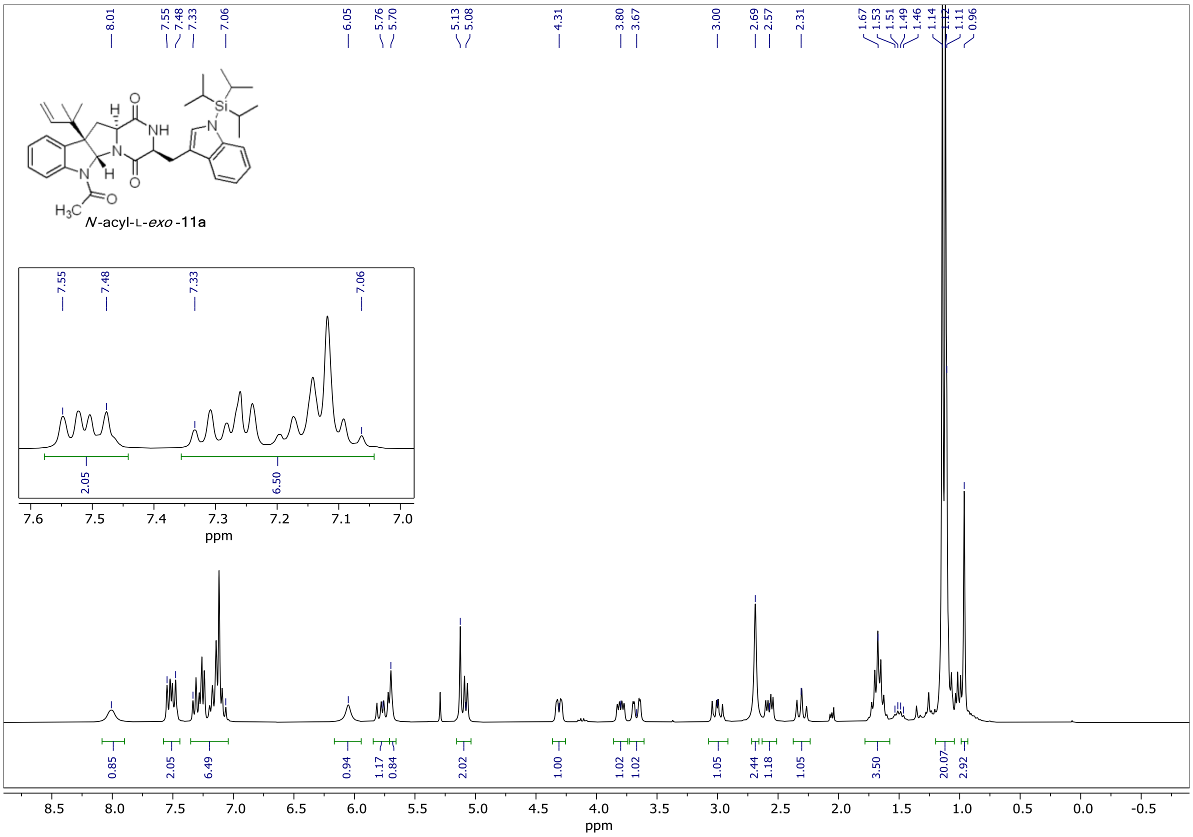

Spectrum 9. ^{1}H-NMR (300 MHz, $CDCl_3$) of *N*-acyl-L-*exo*-**11a**.

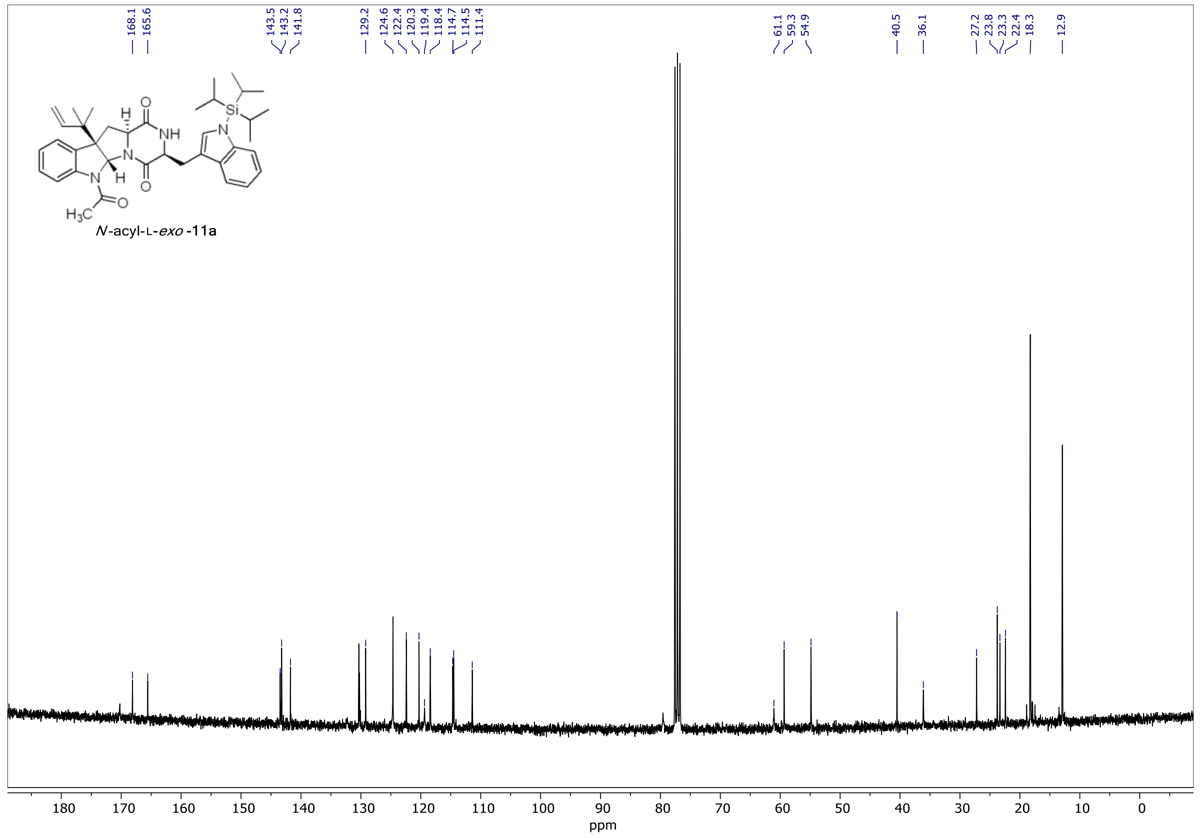

Spectrum 10. ^{13}C-NMR (75 MHz, $CDCl_3$) of *N*-acyl-L-*exo*-**11a**.

2.5 Synthesis of (3*S*,5a*S*,10b*S*,11a*S*)-6-Acetyl-10b-(2-methylbut-3-en-2-yl)-3-((1-(triisopropylsilyl)-1*H*-indol-3-yl)methyl)-2,3,6,10b,11,11a-hexahydro-4*H*-pyrazino[1',2':1,5]pyrrolo[2,3-*b*]indole-1,4(5a*H*)-dione (*N*-acyl-L-*endo*-11b)

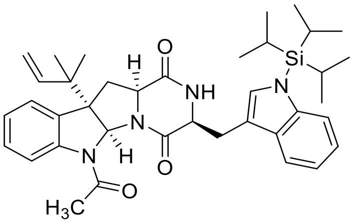

N-acyl-L-*endo*-**11b**

$C_{38}H_{50}N_4O_3Si$

638.93 g/mol

L-*endo*-**11b** (40.8 mg, 69.4 µmol, 1.0 eq.) was dissolved in dichloromethane (1 mL), triethylamine (11.3 µL, 8.3 mg, 82 µmol, 1.2 eq.) and acetyl chloride (12.2 µL, 13.4 mg, 0.17 mmol, 2.5 eq.) were added and the reaction mixture was stirred for 16 h. Then, more triethylamine (50 µL, 37 mg, 0.37 mmol, 5.3 eq.) was added and the solvent was removed *in vacuo*. The crude product was purified *via* column chromatography (PE/EtOAc = 1:1). *N*-acyl-L-*endo*-**11b** (30.7 mg, 48.0 µmol, 69 %) was obtained as a white solid.

R*f*: 0.44 (PE/EtOAc = 1:2). **Mp.**: 128-129 °C. $[\alpha]_D^{20}$ = - 89.0 ° (*c* = 1.0, $CHCl_3$). **^{1}H-NMR** (400 MHz, $CDCl_3$): δ = 7.98 (s, 1H), 7.50 – 7.44 (m, 2H), 7.32 – 7.26 (m, 2H), 7.16 (ddd, ^3J = 8.2 Hz, ^3J = 7.0 Hz, ^4J = 1.4 Hz, 1H), 7.14

– 7.07 (m, 2H), 7.04 (s, 1H), 5.82 (dd, $^3J_{trans}$ = 17.3 Hz, $^3J_{cis}$ = 10.9 Hz, 1H), 5.38 (s, 1H), 5.15 (d, $^3J_{cis}$ = 10.6 Hz, 1H), 5.12 (d, $^3J_{trans}$ = 17.1 Hz, 1H), 4.20 (dd, ^3J = 11.2 Hz, ^3J = 3.6 Hz, 1H), 4.17 – 4.09 (m, 1H), 3.66 (dd, ^2J = 15.2 Hz, ^3J = 3.6 Hz, 1H), 2.93 – 2.80 (m, 1H), 2.75 (dd, ^2J = 15.0 Hz, ^3J = 11.0 Hz, 1H), 2.65 (dd, ^2J = 13.5 Hz, ^3J = 10.1 Hz, 1H), 2.59 (s, 3H), 1.65 (sept, ^3J = 7.5 Hz, 3H), 1.16 – 1.07 (m, 21H), 0.92 (s, 3H) ppm. **^{13}C-NMR** (101 MHz, $CDCl_3$): δ = 171.1, 168.6, 168.2, 143.4, 143.3, 141.9, 141.5, 130.2, 130.0, 128.8, 124.4, 122.3, 120.1, 118.3, 114.8, 114.5, 111.8, 58.1, 55.1, 41.1, 26.4, 24.2, 22.8, 22.3, 18.3, 12.9 ppm. **FT-IR** (ATR): $\tilde{\nu}$ = 2946, 2867, 1682, 1373, 1317, 1142, 1014, 962, 922, 882, 740, 687, 650, 516 cm^{-1}. **HR-MS** (ESI) *m/z*: $[M+H]^+$ calc. for $C_{38}H_{51}N_4O_3Si^+$ = 639.3725, found = 639.3717.

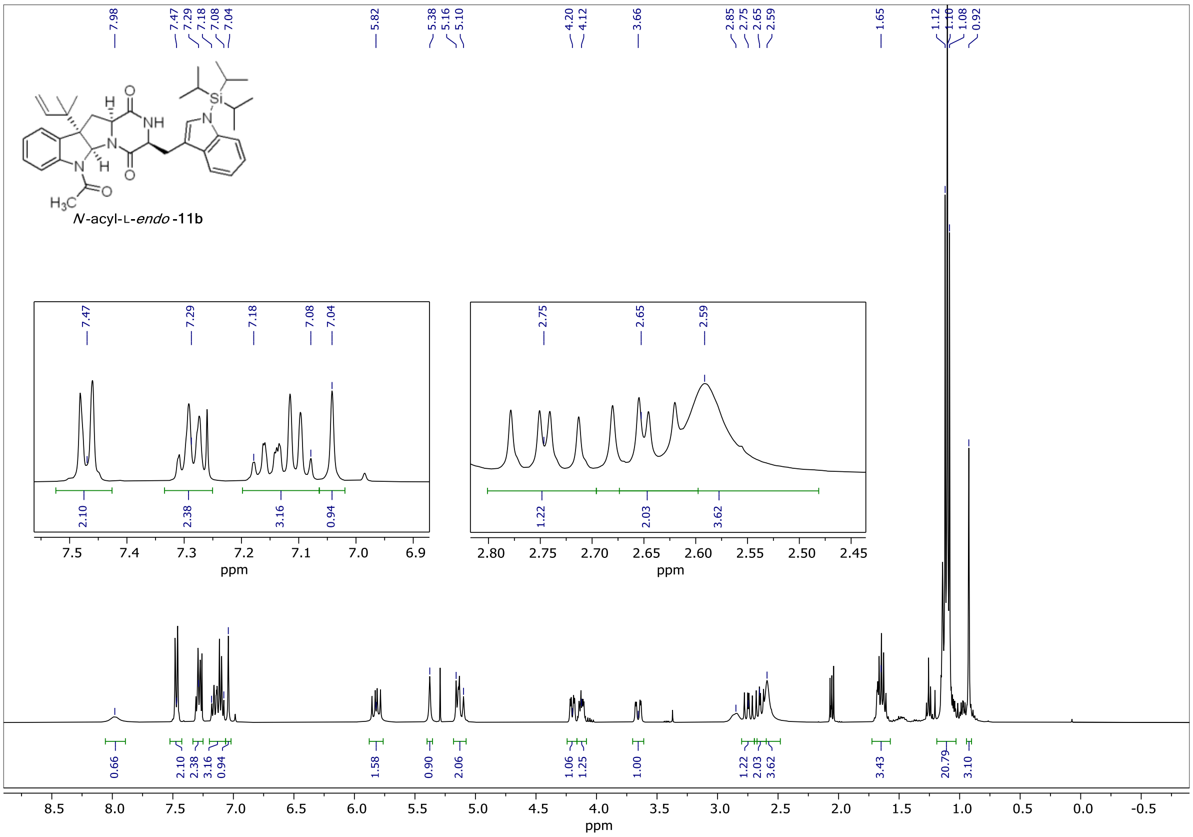

Spectrum 11. ^{1}H-NMR (400 MHz, $CDCl_3$) of *N*-acyl-L-*endo*-**11b**.

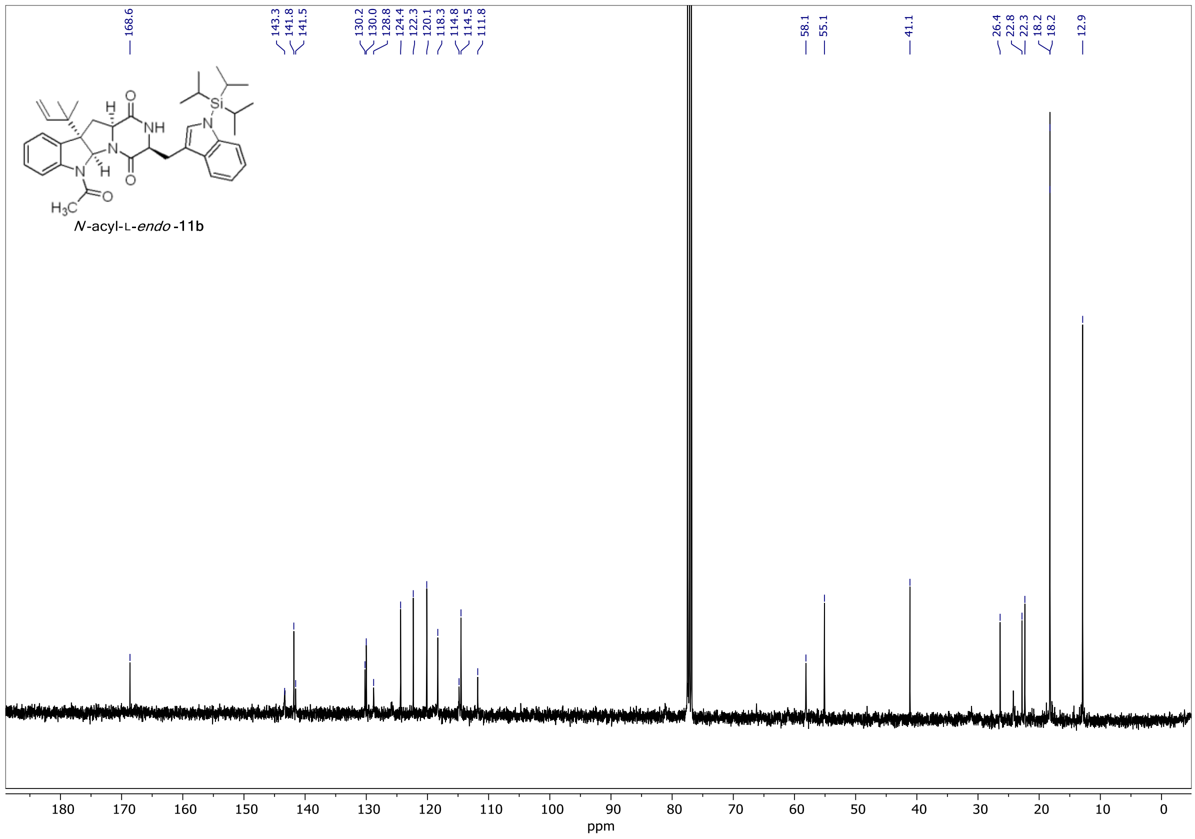

Spectrum 12. ^{13}C-NMR (101 MHz, $CDCl_3$) of *N*-acyl-L-*endo*-**11b**.

2.6 Synthesis of (3*S*,5a*S*,10b*R*,11a*S*)-3-((1*H*-Indol-3-yl)methyl)-10b-(2-methylbut-3-en-2-yl)-2,3,6,10b,11,11a-hexahydro-4*H*-pyrazino[1',2':1,5]pyrrolo[2,3-*b*]indole-1,4(5a*H*)-dione (L-*exo*-5a)

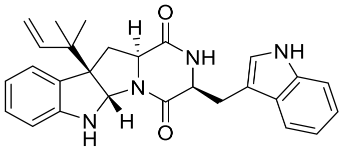

L-*exo*-**5a**

$C_{27}H_{28}N_4O_2$

440.55 g/mol

L-*exo*-**11a** (66.6 mg, 0.112 mmol, 1.0 eq.) was dissolved in THF (1 mL) and TBAF (122 µL, 0.122 mmol, 1.1 eq., 1.0 M solution in THF) was added. After stirring for 1.5 h, the solvent was removed *in vacuo* and the crude product was purified *via* column chromatography (PE/EtOAc = 1:1). L-*exo*-**5a** (49.0 mg, 0.111 mmol, 99 %) was obtained as a white solid.

R*f*: 0.27 (PE/EtOAc = 1:2). **Mp.**: 105-106 °C. $[\alpha]_D^{20}$ = - 280.7 ° (*c* = 1.0, $CHCl_3$). **^{1}H-NMR** (600 MHz, $CDCl_3$): δ = 8.19 (s, 1H), 7.55 (dd, ^3J = 7.9 Hz, ^4J = 1.1 Hz, 1H), 7.38 (dt, ^3J = 8.3 Hz, ^4J = 0.9 Hz, 1H), 7.22 (ddd, ^3J = 8.2 Hz, ^3J = 7.0 Hz, ^4J = 1.1 Hz, 1H), 7.16 – 7.09 (m, 3H), 7.08 (d, ^3J = 2.4 Hz, 1H), 6.75 (dt, ^3J = 7.5 Hz, ^4J = 1.0 Hz, 1H), 6.61 (d, ^3J = 7.8 Hz, 1H), 5.97 (dd, $^3J_{trans}$ = 17.4 Hz, $^3J_{cis}$ = 10.8 Hz, 1H), 5.69 (s, 1H), 5.55 (s, 1H), 5.12 (dd, $^3{}_{cis}$ = 10.8 Hz, ^5J = 1.1 Hz, 1H), 5.08 (dd, $^3J_{trans}$ = 17.4 Hz, ^5J = 1.2 Hz, 1H), 5.04 (s, 1H), 4.30 (ddd, ^3J =

10.9 Hz, ^{3}J = 3.7 Hz, ^{5}J = 1.9 Hz, 1H), 3.91 (ddd, ^{3}J = 11.2 Hz, ^{3}J = 6.2 Hz, ^{5}J = 1.9 Hz, 1H), 3.74 (ddd, ^{2}J = 15.1 Hz, ^{3}J = 3.7 Hz, ^{4}J = 1.1 Hz, 1H), 2.97 (dd, ^{2}J = 15.1 Hz, ^{3}J = 10.9 Hz, 1H), 2.51 (dd, ^{2}J = 12.6 Hz, ^{3}J = 6.3 Hz, 1H), 2.41 (dd, ^{2}J = 12.6 Hz, ^{3}J = 11.2 Hz, 1H), 1.11 (s, 3H), 1.01 (s, 3H) ppm. **^{13}C-NMR** (151 MHz, $CDCl_3$): δ = 169.1, 166.1, 150.1, 143.6, 136.8, 129.1, 129.0, 126.7, 125.2, 123.4, 123.0, 120.2, 119.1, 118.6, 114.6, 111.7, 109.8, 109.4, 77.9, 61.8, 59.2, 54.8, 41.0, 36.1, 27.2, 23.0, 22.6 ppm. **FT-IR** (ATR): $\tilde{\nu}$ = 3306, 2925, 2864, 1651, 1456, 1416, 1315, 1213, 1081, 1059, 1009, 918, 881, 834, 740, 675, 425 cm^{-1}. **HR-MS** (ESI) *m/z*: [M+H]$^{+}$ calc. for $C_{27}H_{29}N_4O_2^{+}$ = 441.2285, found = 441.2292.

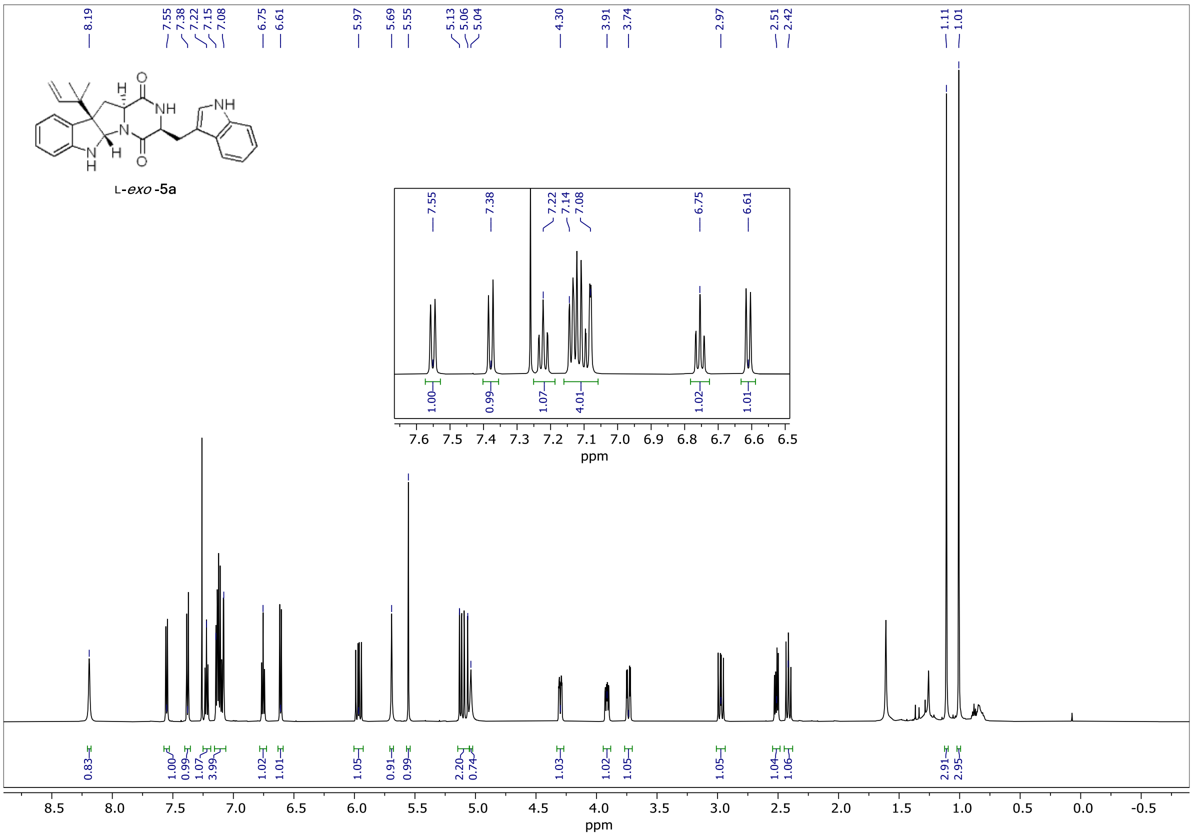

Spectrum 13. ^{1}H-NMR (600 MHz, $CDCl_3$) of L-*exo*-**5a**.

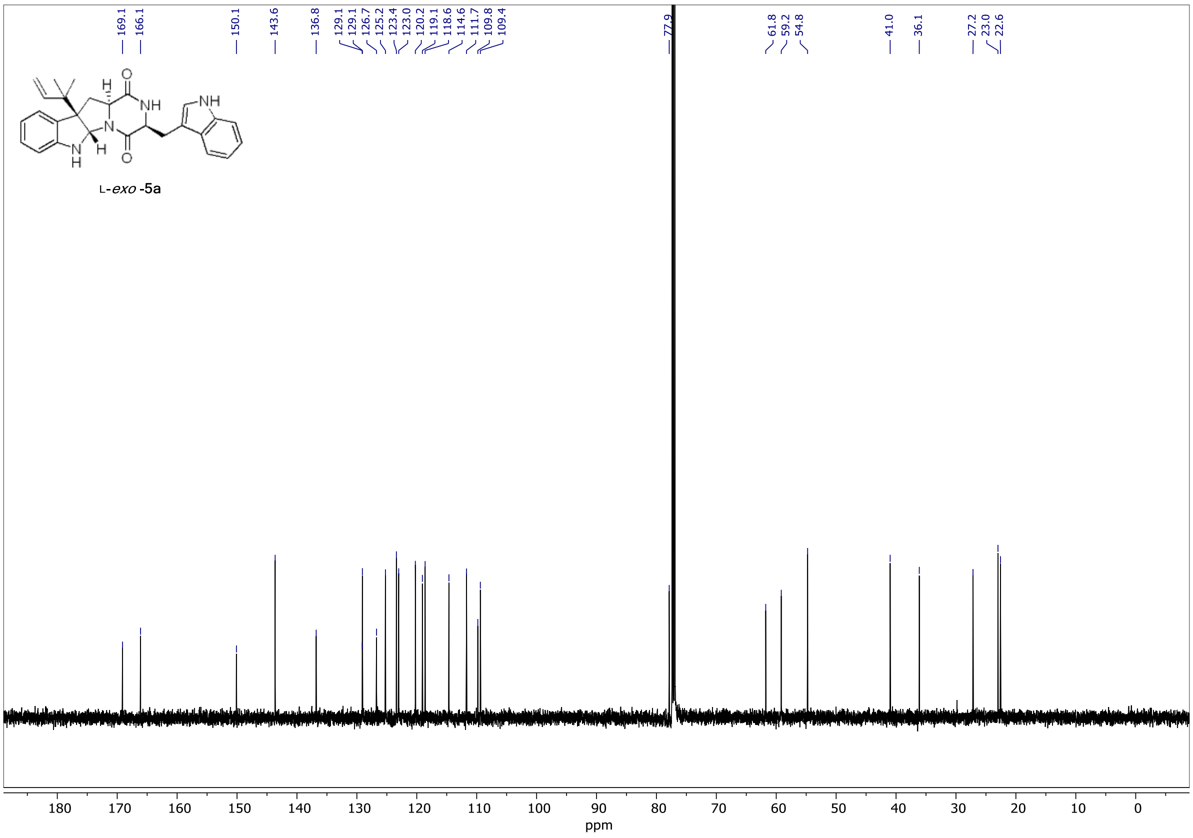

Spectrum 14. ^{13}C-NMR (151 MHz, $CDCl_3$) of L-*exo*-**5a**.

2.7 Synthesis of (3*S*,5a*R*,10b*S*,11a*S*)-3-((1*H*-Indol-3-yl)methyl)-10b-(2-methylbut-3-en-2-yl)-2,3,6,10b,11,11a-hexahydro-4*H*-pyrazino[1',2':1,5]pyrrolo[2,3-*b*]indole-1,4(5a*H*)-dione (L-*endo*-5b)

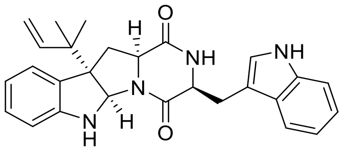

L-*endo*-**5b**

$C_{27}H_{28}N_4O_2$

440.55 g/mol

L-*endo*-**11b** (32.0 mg, 53.6 µmol, 1.0 eq.) was dissolved in THF (1 mL) and TBAF (59 µL, 0.59 mmol, 1.1 eq., 1.0 M solution in THF, buffered with AcOH (59 µL, 62 mg, 1.0 mmol) to prevent epimerization) was added. After stirring for 1.5 h, the solvent was removed *in vacuo* and the crude product was purified *via* column chromatography (PE/EtOAc = 1:1). L-*endo*-**5b** (21.4 mg, 48.6 µmol, 91 %) was obtained as a white solid.

R*f*: 0.35 (PE/EtOAc = 1:2). **Mp.**: 129-130 °C. $[\alpha]_D^{20}$ = + 54.1 ° (*c* = 1.0, $CHCl_3$). ^{1}**H-NMR** (300 MHz, $CDCl_3$): δ = 8.12 (s, 1H), 7.55 (d, ^3J = 7.9 Hz, 1H), 7.38 – 7.05 (m, 5H), 6.94 (d, ^3J = 2.4 Hz, 1H), 6.75 (dt, ^3J = 7.5 Hz, ^4J = 1.1 Hz, 1H), 6.59 (d, ^3J = 7.6 Hz, 1H), 5.94 (dd, $^3J_{trans}$ = 17.3 Hz, $^3J_{cis}$ = 10.9 Hz, 1H), 5.72 (s, 1H), 5.54 (s, 1H), 5.42 (s, 1H), 5.15 (dd, $^3J_{cis}$ = 10.9 Hz, ^5J = 0.8 Hz, 1H), 5.12 (dd, $^3J_{trans}$ = 17.3 Hz, ^5J = 0.8 Hz, 1H), 4.29 (ddd, ^3J

= 10.5 Hz, ^3J = 3.8 Hz, ^5J = 1.7 Hz, 1H), 4.13 – 3.99 (m, 1H), 3.74 – 3.61 (m, 1H), 2.90 (dd, ^2J = 14.9 Hz, ^3J = 10.5 Hz, 1H), 2.78 (dd, ^2J = 13.8 Hz, ^3J = 8.8 Hz, 1H), 2.38 (dd, ^2J = 13.8 Hz, ^3J = 9.2 Hz, 1H), 1.14 (s, 3H), 0.99 (s, 3H) ppm. **^{13}C-NMR** (75 MHz, $CDCl_3$): δ = 168.9, 168.0, 148.6, 143.6, 136.7, 131.7, 128.5, 126.7, 125.9, 123.5, 122.9, 120.1, 118.9, 118.6, 114.9, 111.7, 109.6, 109.1, 79.8, 61.8, 57.9, 55.1, 41.8, 36.5, 27.2, 22.6 ppm. **FT-IR** (ATR): $\tilde{\nu}$ = 3357, 2965, 2926, 1660, 1457, 1413, 1339, 1311, 1093, 1010, 921, 741, 466 cm^{-1}. **HR-MS** (ESI) *m*/*z*: $[M+H]^+$ calc. for $C_{27}H_{29}N_4O_2^+$ = 441.2285, found = 441.2267.

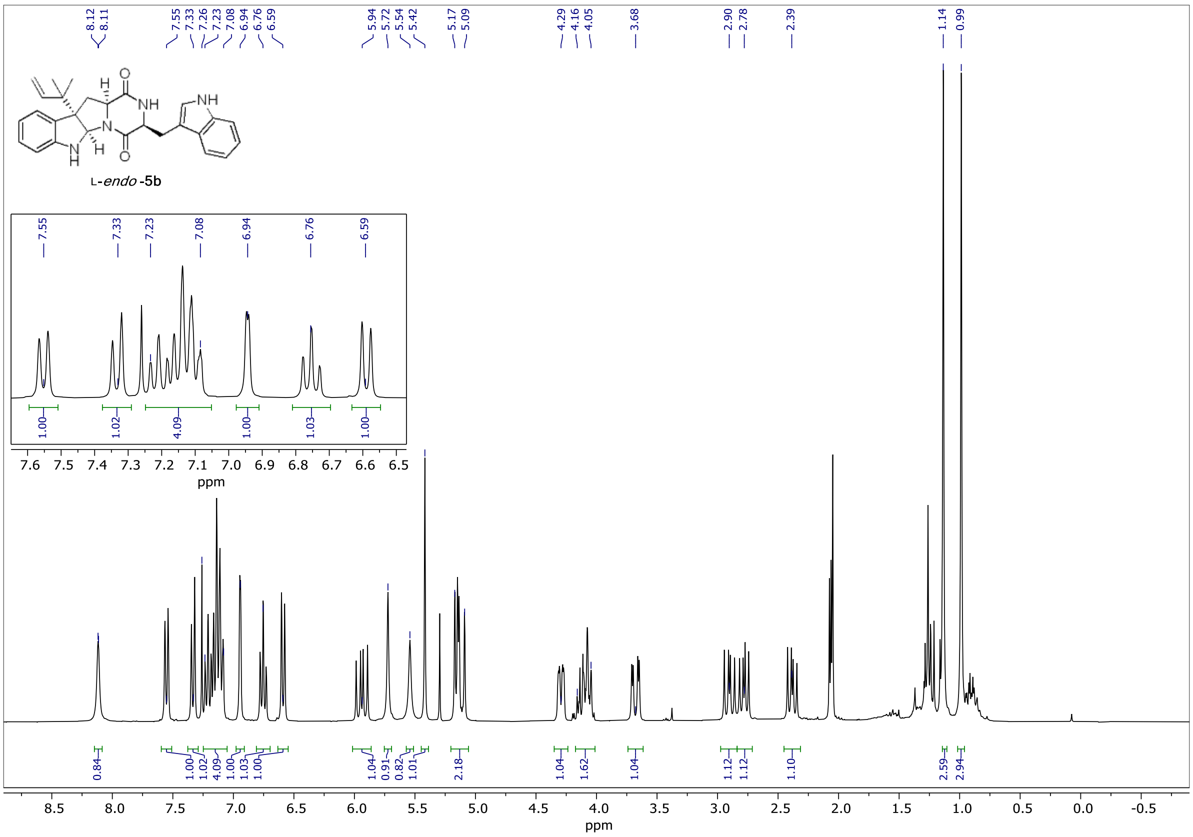

Spectrum 15. ^{1}H-NMR (300 MHz, $CDCl_3$) of L-*endo*-**5b**.

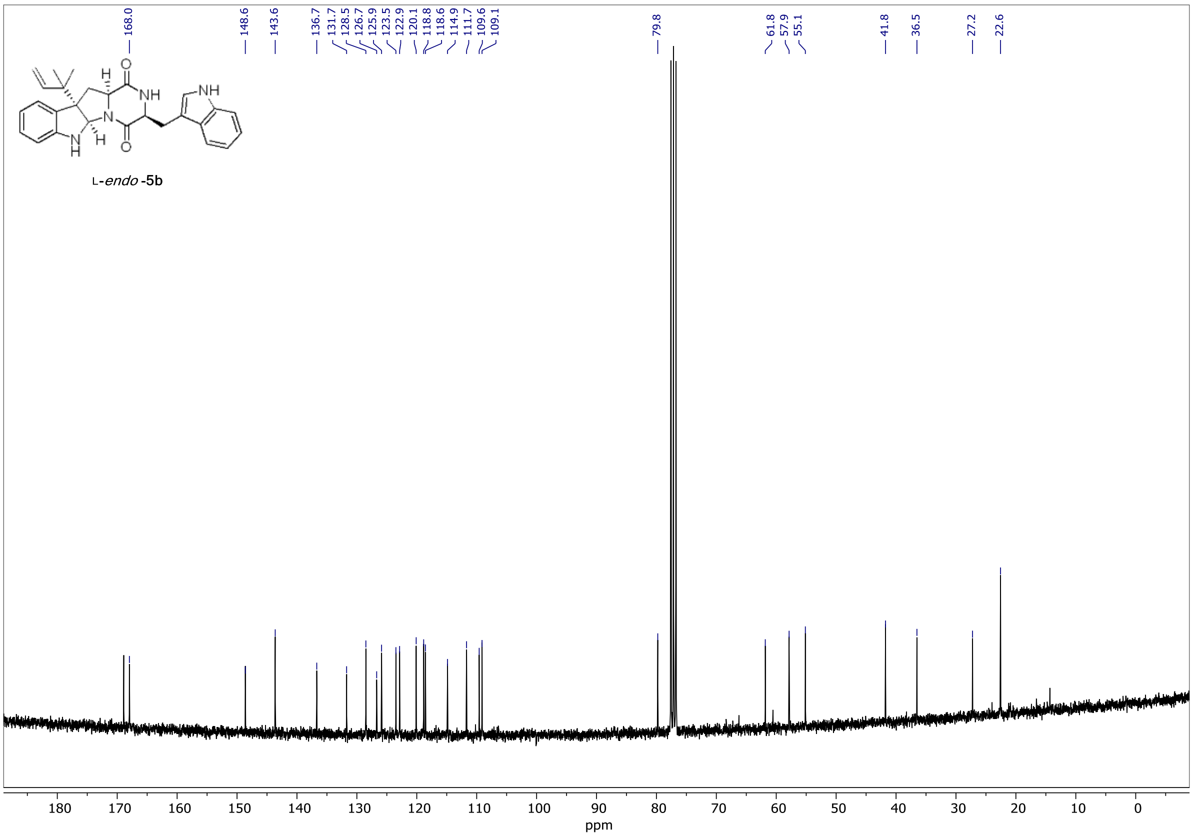

Spectrum 16. ^{13}C-NMR (75 MHz, $CDCl_3$) of L-*endo*-**5b**.

2.8 Synthesis of L-*exo*-Okaramine M (4a)

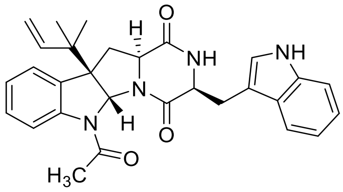

4a

$C_{29}H_{30}N_4O_3$

482.58 g/mol

L-*exo*-**5a** (20.9 mg, 32.7 µmol, 1.0 eq.) was dissolved in THF (0.5 mL) and TBAF (45 µL, 0.45 mmol, 1.4 eq., 1.0 M solution in THF, buffered with AcOH (45 µL, 47 mg, 0.78 mmol) was added. After stirring for 1.5 h, the solvent was removed *in vacuo* and the crude product was purified *via* column chromatography (PE/EtOAc = 1:2). L-*exo*-Okaramine M (**4a**) (15.4 mg, 31.9 µmol, 98 %) was obtained as a white solid.

R*f*: 0.18 (PE/EtOAc = 1:3). **Mp.**: 171-172 °C. $[\alpha]_D^{20}$ = - 124.9 ° (*c* = 1.0, $CHCl_3$), - 125.6 ° (*c* = 1.0, CH_3OH). **^{1}H-NMR** (400 MHz, acetone-d_6): δ = 10.18 (s, 1H), 7.97 (d, ^3J = 8.3 Hz, 1H), 7.65 (dd, ^3J = 7.9 Hz, ^4J = 1.2 Hz, 1H), 7.40 (dt, ^3J = 8.2 Hz, ^4J = 1.0 Hz, 1H), 7.36 (dd, ^3J = 7.5 Hz, ^4J = 1.4 Hz, 1H), 7.31 – 7.22 (m, 2H), 7.11 (ddt, ^3J = 8.2 Hz, ^3J = 7.0 Hz, ^4J = 1.3 Hz, 2H), 7.02 (ddd, ^3J = 8.0 Hz, ^3J = 7.0 Hz, ^4J = 1.1 Hz, 1H), 6.82 (s, 1H), 5.97 (s, 1H), 5.67 (dd, $^3J_{trans}$ = 17.4 Hz, $^3J_{cis}$ = 10.7 Hz, 1H), 5.02 (dd, $^3J_{trans}$ = 17.4 Hz, ^4J = 1.0 Hz, 1H), 4.99 (dd, $^3J_{cis}$

= 10.8 Hz, ^{4}J = 1.1 Hz, 1H), 4.48 (ddt, ^{3}J = 6.6 Hz, ^{3}J = 4.2 Hz, ^{4}J = 1.2 Hz, 1H), 3.70 (ddd, ^{3}J = 11.7 Hz, ^{3}J = 5.4 Hz, ^{4}J = 1.5 Hz, 1H), 3.40 (ddd, ^{2}J = 14.8 Hz, ^{3}J = 4.1 Hz, ^{4}J = 1.0 Hz, 1H), 3.27 (dd, ^{2}J = 14.8 Hz, ^{3}J = 6.7 Hz, 1H), 2.62 (s, 3H), 2.36 (dd, ^{2}J = 12.5 Hz, ^{3}J = 5.5 Hz, 1H), 1.86 (t, ^{3}J = 12.0 Hz, 1H), 0.93 (s, 3H), 0.82 (s, 3H) ppm. **^{13}C-NMR** (101 MHz, acetone-*d*$_6$): δ = 170.3, 167.7, 166.7, 144.9, 144.5, 137.7, 133.6, 129.3, 128.6, 125.7, 125.4, 124.8, 122.4, 119.8, 119.7, 119.1, 114.3, 112.2, 110.1, 80.1, 61.8, 59.9, 56.4, 41.0, 37.3, 28.3, 24.2, 23.5, 22.5 ppm. **FT-IR** (ATR): $\tilde{\nu}$ = 3292, 2967, 1667, 1477, 1414, 1385, 1337, 1309, 1279, 1103, 1009, 921, 740, 423 cm^{-1}. **HR-MS** (ESI) *m*/*z*: [M+H]$^+$ calc. for $C_{29}H_{31}N_4O_3^+$ = 483.2391, found = 483.2411.

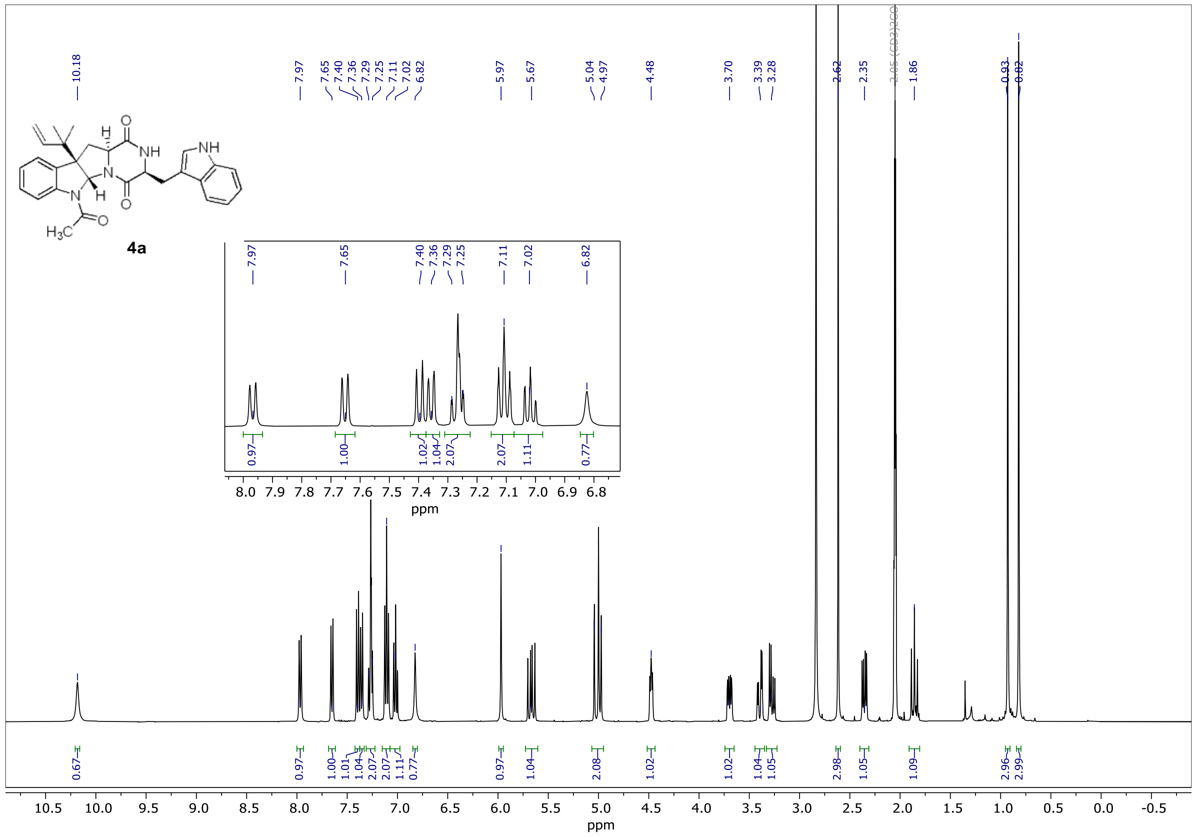

Spectrum 17. ^{1}H-NMR (400 MHz, acetone-*d*$_6$) of **4a**.

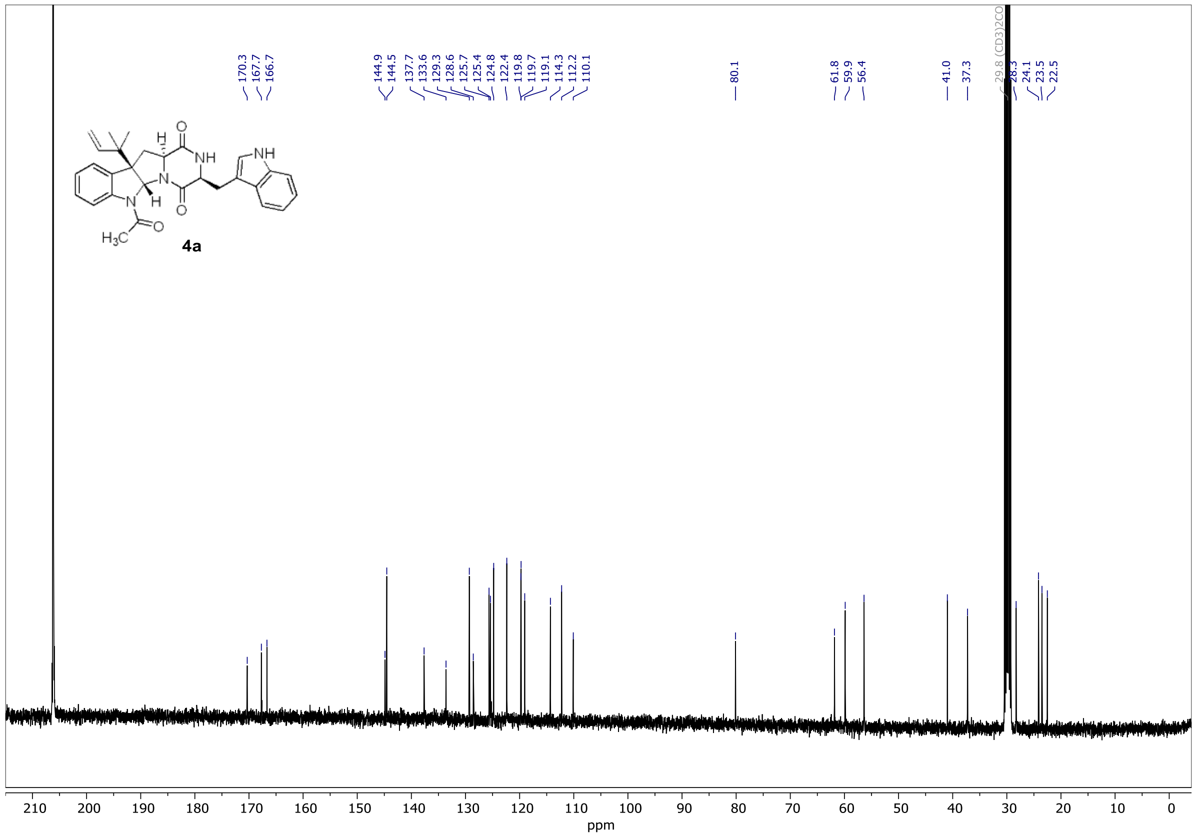

Spectrum 18. ^{13}C-NMR (101 MHz, acetone-d_6) of **4a**.

2.9 Synthesis of L-*endo*-Okaramine M (4b)

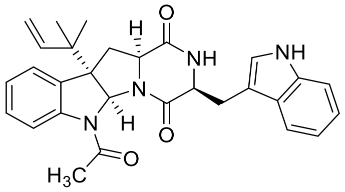

4b

$C_{29}H_{30}N_4O_3$

482.58 g/mol

L-*endo*-**5b** (15.6 mg, 24.4 µmol, 1.0 eq.) was dissolved in THF (0.5 mL) and TBAF (45 µL, 0.45 mmol, 1.8 eq., 1.0 M solution in THF, buffered with AcOH (45 µL, 47 mg, 0.78 mmol) to prevent epimerization) was added. After stirring for 1.5 h, the solvent was removed *in vacuo* and the crude product was purified *via* column chromatography (PE/EtOAc = 1:2). L-*endo*-Okaramine M (**4b**) (11.0 mg, 22.8 µmol, 93 %) was obtained as a white solid.

R*f*: 0.15 (PE/EtOAc = 1:3). **Mp.**: 173-174 °C. $[\alpha]_D^{20}$ = - 110.7 ° (c = 1.0, $CHCl_3$). **^{1}H-NMR** (400 MHz, acetone-d_6): δ = 10.00 (s, 1H), 7.92 (s, 1H), 7.52 (d, ^3J = 7.9 Hz, 1H), 7.38 – 7.29 (m, 2H), 7.24 (dt, ^3J = 7.7 Hz, ^4J = 1.3 Hz, 1H), 7.13 (d, ^4J = 2.4 Hz, 1H), 7.11 – 7.03 (m, 2H), 6.99 (ddd, ^3J = 8.0 Hz, ^3J = 7.0 Hz, ^4J = 1.0 Hz, 1H), 6.18 (s, 1H), 5.98 (s, 1H), 5.92 (dd, $^3J_{trans}$ = 17.4 Hz, $^3J_{cis}$ = 10.8 Hz, 1H), 5.14 (dd, $^3J_{trans}$ = 17.4 Hz, ^5J = 1.2 Hz, 1H), 5.10

(dd, $^{3}J_{cis}$ = 10.8 Hz, ^{5}J = 1.2 Hz, 1H), 4.38 (dd, ^{3}J = 10.2 Hz, ^{3}J = 5.2 Hz, 1H), 4.31 (dd, ^{3}J = 8.6 Hz, ^{3}J = 4.0 Hz, 1H), 3.47 (ddd, ^{2}J = 15.2 Hz, ^{3}J = 4.0 Hz, ^{4}J = 1.0 Hz, 1H), 2.87 (dd, ^{2}J = 15.4 Hz, ^{3}J = 8.5 Hz, 1H), 2.76 (dd, ^{2}J = 13.6 Hz, ^{3}J = 5.1 Hz, 1H), 2.67 (dd, ^{2}J = 13.6 Hz, ^{3}J = 10.2 Hz, 1H), 2.57 (s, 3H), 1.15 (s, 3H), 0.95 (s, 3H) ppm. **^{13}C-NMR** (101 MHz, acetone-d_6): δ = 170.1, 169.6, 169.2, 144.8, 142.9, 137.7, 129.0, 128.3, 126.8, 124.8, 124.4, 122.3, 119.7, 119.1, 118.9, 114.4, 112.3, 110.4, 81.5, 61.8, 58.6, 56.4, 41.8, 31.4, 26.0, 24.7, 23.3, 22.4 ppm. **FT-IR** (ATR): $\tilde{\nu}$ = 3294, 2912, 2852, 1667, 1476, 1459, 1378, 1320, 1286, 1181, 1097, 1010, 924, 739, 561, 462, 425 cm^{-1}. **HR-MS** (ESI) *m*/*z*: [M+H]$^{+}$ calc. for $C_{29}H_{31}N_4O_3^{+}$ = 483.2391, found = 483.2403.

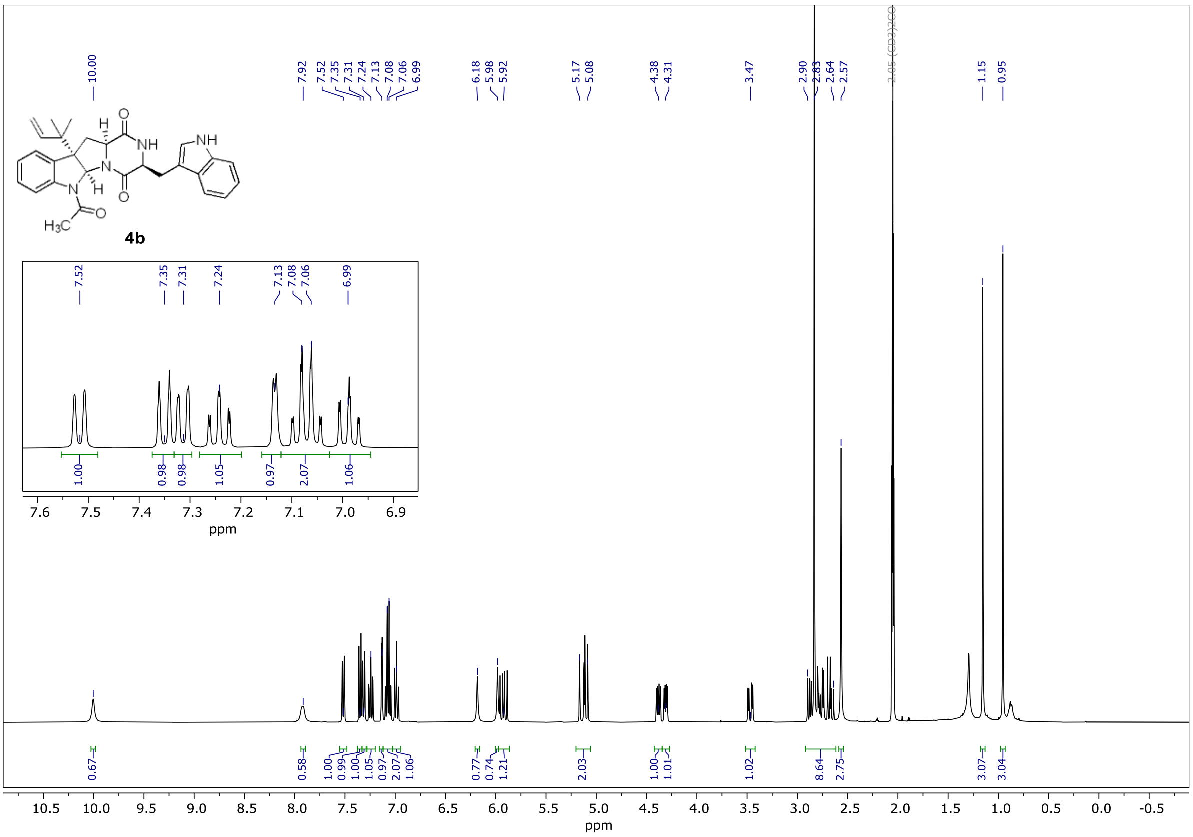

Spectrum 19. ^{1}H-NMR (400 MHz, acetone-d_6) of **4b**.

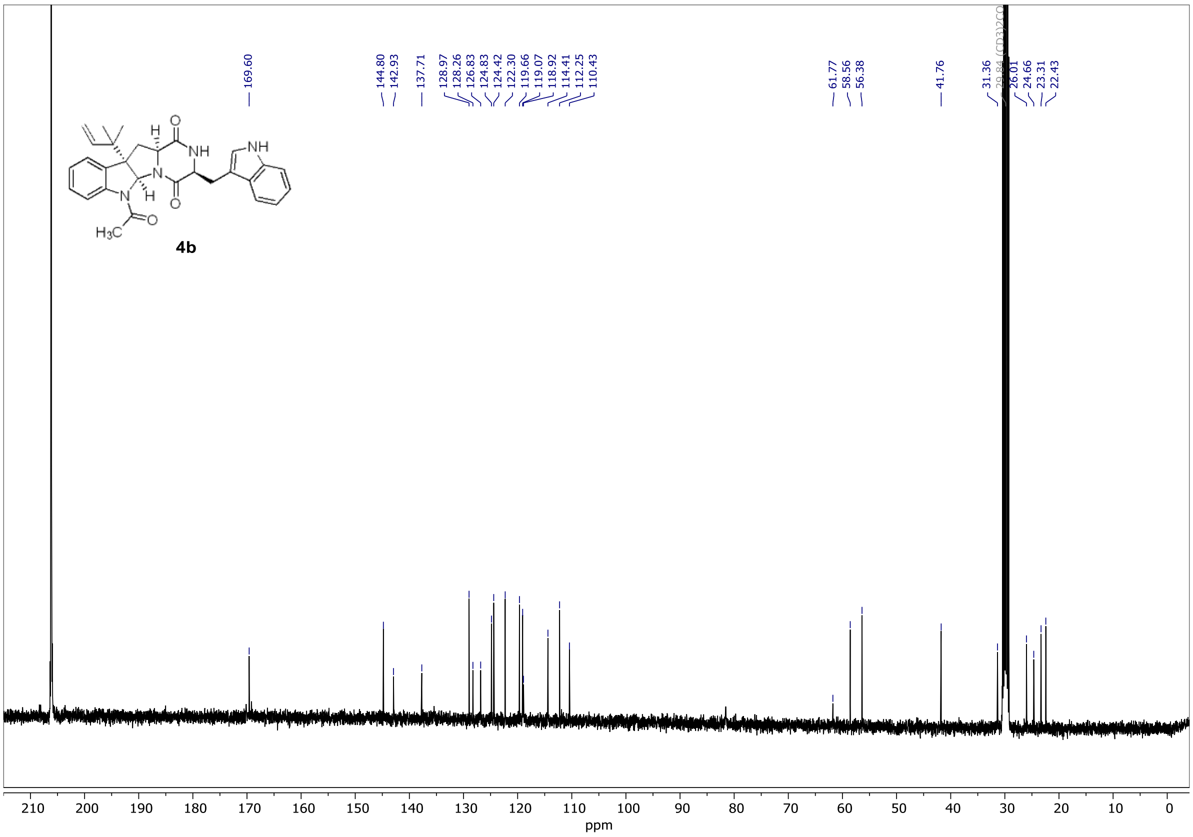

Spectrum 20. ^{13}C-NMR (101 MHz, acetone-d_6) of **4b**.

2.10 Synthesis of D-*exo*-Okaramine M (4c)

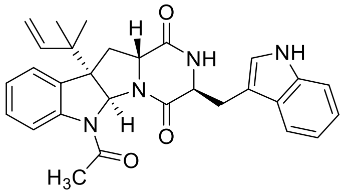

4c

$C_{29}H_{30}N_4O_3$

482.58 g/mol

L-*endo*-Okaramine M (**4b**) (9.0 mg, 19 µmol, 1.0 eq.) was dissolved in THF (0.5 mL) and TBAF (36 µL, 36 µmol, 1.9 eq., 1.0 M solution in THF) was added. After stirring for 1.5 h, the solvent was removed *in vacuo* and the crude product was purified *via* column chromatography (PE/EtOAc = 1:2). D-*exo*-Okaramine M (**4c**) (5.0 mg, 10 µmol, 53 %) was obtained as a white solid.

^{1}H-NMR (600 MHz, acetone-d_6): δ = 9.71 (s, 1H), 7.88 (d, ^3J = 8.0 Hz, 1H), 7.42 (dt, ^3J = 7.9 Hz, ^4J = 1.0 Hz, 1H), 7.35 (d, ^4J = 3.3 Hz, 1H), 7.29 – 7.25 (m, 1H), 7.23 – 7.20 (m, 1H), 7.15 (dt, ^3J = 8.2 Hz, ^4J = 0.9 Hz, 1H), 7.05 (dt, ^3J = 7.5 Hz, ^4J = 1.1 Hz, 1H), 6.99 (ddd, ^3J = 8.2 Hz, ^3J = 7.0 Hz, ^4J = 1.2 Hz, 1H), 6.93 (ddd, ^3J = 8.0 Hz, ^3J = 6.9 Hz, ^4J = 1.0 Hz, 1H), 6.87 (d, ^3J = 2.4 Hz, 1H), 6.01 (s, 1H), 5.86 (dd, $^3J_{trans}$ = 17.4 Hz, $^3J_{cis}$ = 10.8 Hz, 1H), 5.11 (dd, $^3J_{trans}$ = 17.4 Hz, ^5J = 1.2 Hz, 1H), 5.05 (dd, $^3J_{cis}$ = 10.8 Hz, ^5J = 1.1 Hz, 1H), 4.20 (dt, ^3J = 4.8 Hz,

^3J = 3.5 Hz, 1H), 3.22 (dd, ^2J = 14.5 Hz, ^3J = 5.0 Hz, 1H), 3.15 – 3.10 (m, 1H), 2.80 (s, 1H), 2.64 (s, 3H), 2.60 (dd, ^2J = 11.7 Hz, ^3J = 5.5 Hz, 1H), 2.26 (dd, ^2J = 12.5 Hz, ^3J = 5.5 Hz, 1H), 2.16 – 2.08 (m, 1H), 1.10 (s, 3H), 0.93 (s, 3H) ppm. **^{13}C-NMR** (126 MHz, acetone-d_6): δ = 170.1, 167.9, 166.9, 144.8, 144.5, 137.0, 133.3, 129.1, 128.4, 125.2, 125.0, 124.6, 122.2, 120.0, 119.8, 119.1, 114.3, 111.9, 109.1, 80.1, 61.1, 59.3, 58.9, 41.0, 37.3, 31.2, 24.0, 23.6, 22.7 ppm. **HR-MS** (ESI) *m/z*: [M+H]$^+$ calc. for $C_{29}H_{31}N_4O_3^+$ = 483.2391, found = 483.2406.

The analytical data is in accordance with the data reported by Iizuka *et al*.[3]

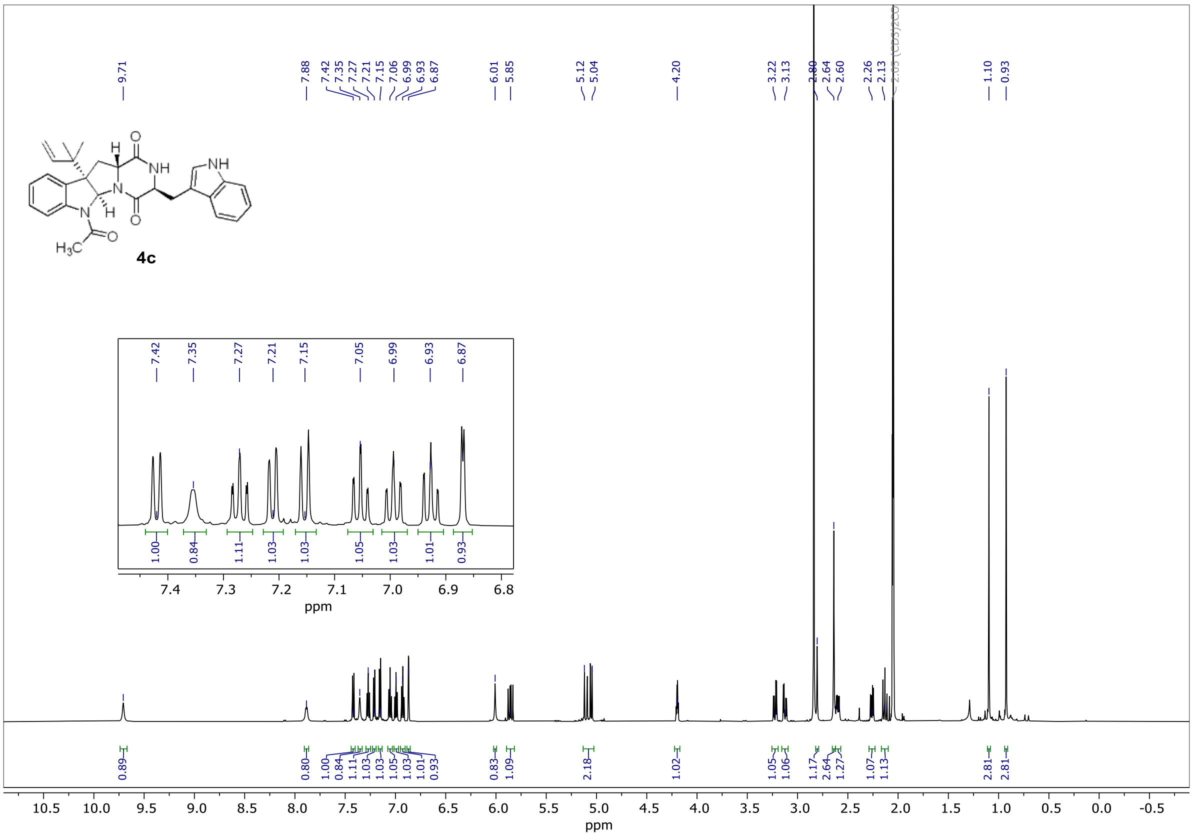

Spectrum 21. ^{1}H-NMR (600 MHz, acetone-d_6) of **4c**.

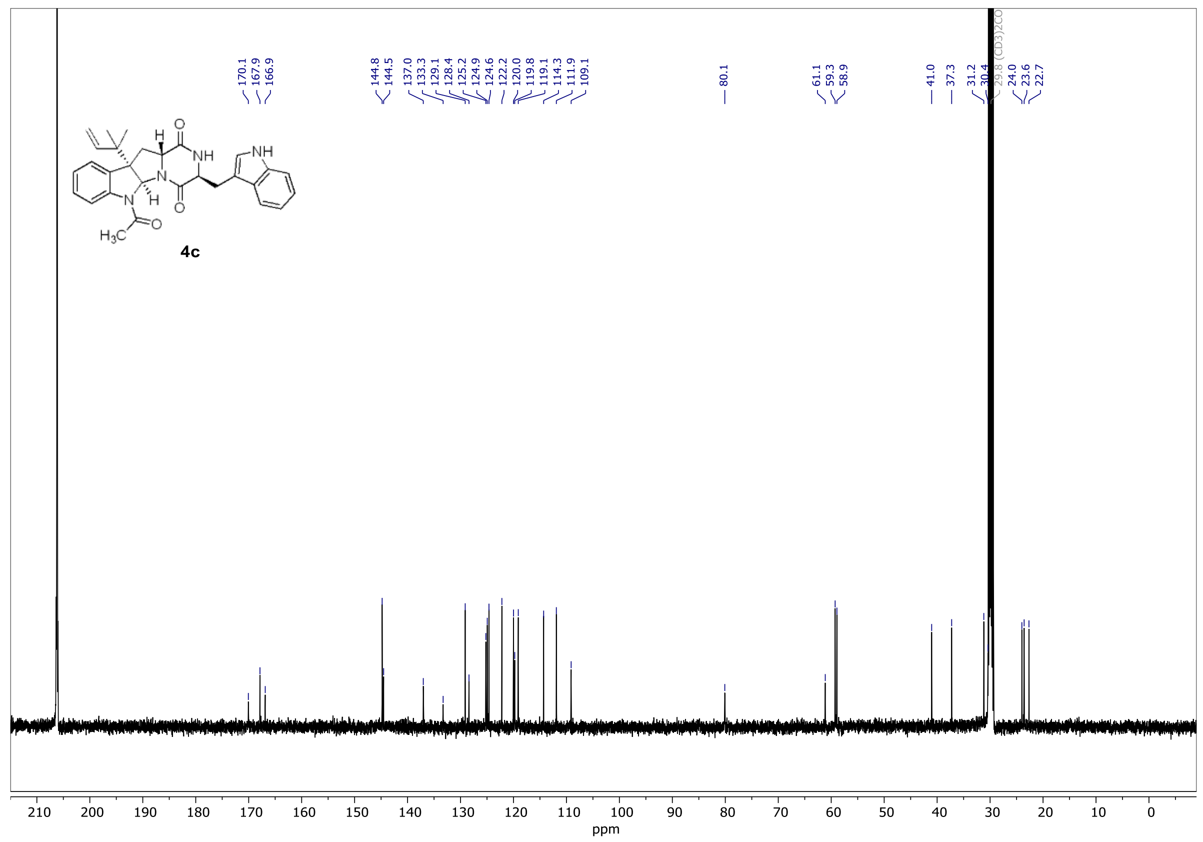

Spectrum 22. ^{13}C-NMR (126 MHz, acetone-d_6) of **4c**.

2.11 Synthesis of Amauromine (1a), Epiamauromine (1b) and Novoamauromine (1c)

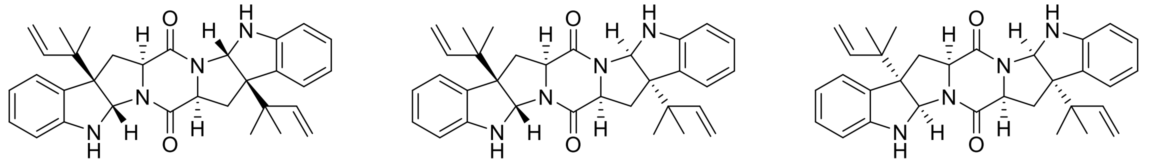

(-)-Amauromine	(-)-Epiamauromine	(+)-Novoamauromine
(**1a**)	(**1b**)	(**1c**)

$C_{32}H_{36}N_4O_2$

508.67 g/mol

General experimental procedure

The reaction was performed under inert conditions.

pre-Okamauromine (**2**) (1.0 eq.) was dissolved in dichloromethane (12 mL/mmol), triethyl borane (2.2 eq., 1.0 M in hexane) was added and the solution was stirred for 10 min. $[Ir(COD)Cl]_2$ (2.5 mol% ≙ 5 mol% [Ir] or 10 mol% ≙ 20 mol% [Ir]) and L1 (10 mol% or 40 mol%, respectively) were dissolved in dichloromethane (0.2 mL/mol% [Ir]) and stirred for 10 min. upon which a bright red solution was obtained. DBU (20 mol%) was added to the substrate solution and the catalyst solution was added directly afterwards. *tert*-Butyl-(2-methylbut-3-en-2-yl) carbonate (prenyl carbonate) (5.0 eq.) was added and the reaction mixture was stirred for 16 h. After removing the solvent *in vacuo*, the residue was purified *via* column chromatography (PE/EtOAc = 5:1).

Three natural products Amauromine (**1a**), Epiamauromine (**1b**) und Novoamauromine (**1c**) could be separated during column chromatography and were obtained as white solids.

The yields and product ratios under different reaction conditions are listed in Table 1. All reactions were carried out in a 0.250 mmol scale.

Table 1. Screening of reaction conditions for the iridium-catalyzed reverse prenylation of *pre*-Okamauromine (2) with product ratios and yields.

Entry	Solvent	[Ir]	Ligand	Product ratio	Yield
**1**	CH_2Cl_2	5 mol%	L1	1.3 : 2.0 : 1.0	90 %
**2**	CH_2Cl_2	5 mol%	(*S*)-L2	5.0 : 4.0 : 1.0	7 %
**3**	CH_2Cl_2	5 mol%	(*R*)-L2	5.0 : 4.5 : 1.0	8 %
**4**	CH_2Cl_2	20 mol%	(*S*)-L2	5.0 : 5.0 : 1.0	16 %
**5**	CH_2Cl_2	20 mol%	(*R*)-L2	5.0 : 4.0 : 1.0	21 %
**6**	THF/CH_2Cl_2 (1:1)	5 mol%	(*S*)-L2	2.8 : 3.8 : 1.0	55 %

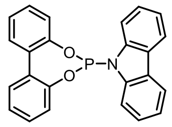

L1

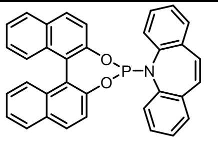

(*S*)-**L2**; (*R*)-**L2**

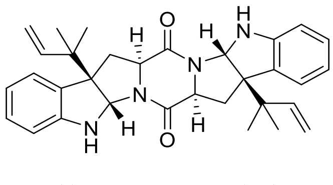

(-)-Amauromine (**1a**)

R*f*: 0.55 (PE/EtOAc = 3:1). **Mp.**: 158-159 °C. $[\alpha]_D^{20}$ = - 525.6 ° (*c* = 1.0, $CHCl_3$). **^{1}H-NMR** (400 MHz, $CDCl_3$): δ = 7.07 (dd, ^3J = 7.6 Hz, ^4J = 1.2 Hz, 2H), 7.02 (dt, ^3J = 7.6 Hz, ^4J = 1.2 Hz, 2H), 6.69 (dt, ^3J = 7.5 Hz, ^4J = 1.0 Hz, 2H), 6.48 (dd, ^3J = 7.8 Hz, ^4J = 1.0 Hz, 2H), 5.99 (dd, $^3J_{trans}$ = 17.4 Hz, $^3J_{cis}$ = 10.8 Hz, 2H), 5.44 (s, 2H), 5.13 (dd, $^3J_{cis}$ = 10.9, ^5J = 1.2 Hz, 2H), 5.07 (dd, $^3J_{trans}$ = 17.4 Hz, ^5J = 1.2 Hz, 2H), 4.94 (s, 2H), 3.84 (dd, ^3J = 9.9 Hz, ^3J = 6.6 Hz, 2H), 2.53 – 2.38 (m, 4H), 1.10 (s, 6H), 1.01 (s, 6H) ppm. **^{13}C-NMR** (101 MHz, $CDCl_3$): δ = 166.6, 150.0, 143.7, 129.1, 129.0, 125.0, 119.0, 114.5, 109.4, 77.4, 62.0, 60.6, 40.9, 35.2, 23.0, 22.7 ppm. **FT-IR** (ATR): $\tilde{\nu}$ = 3340, 2973, 2931, 1747, 1660, 1472, 1440, 1415, 1386, 1369, 1247, 1149, 1008, 840, 741 cm^{-1}. **HR-MS** (ESI) *m*/*z*: $[M+H]^+$ calc. for $C_{32}H_{37}N_4O_2^+$ = 509.2911, found = 509.2911.

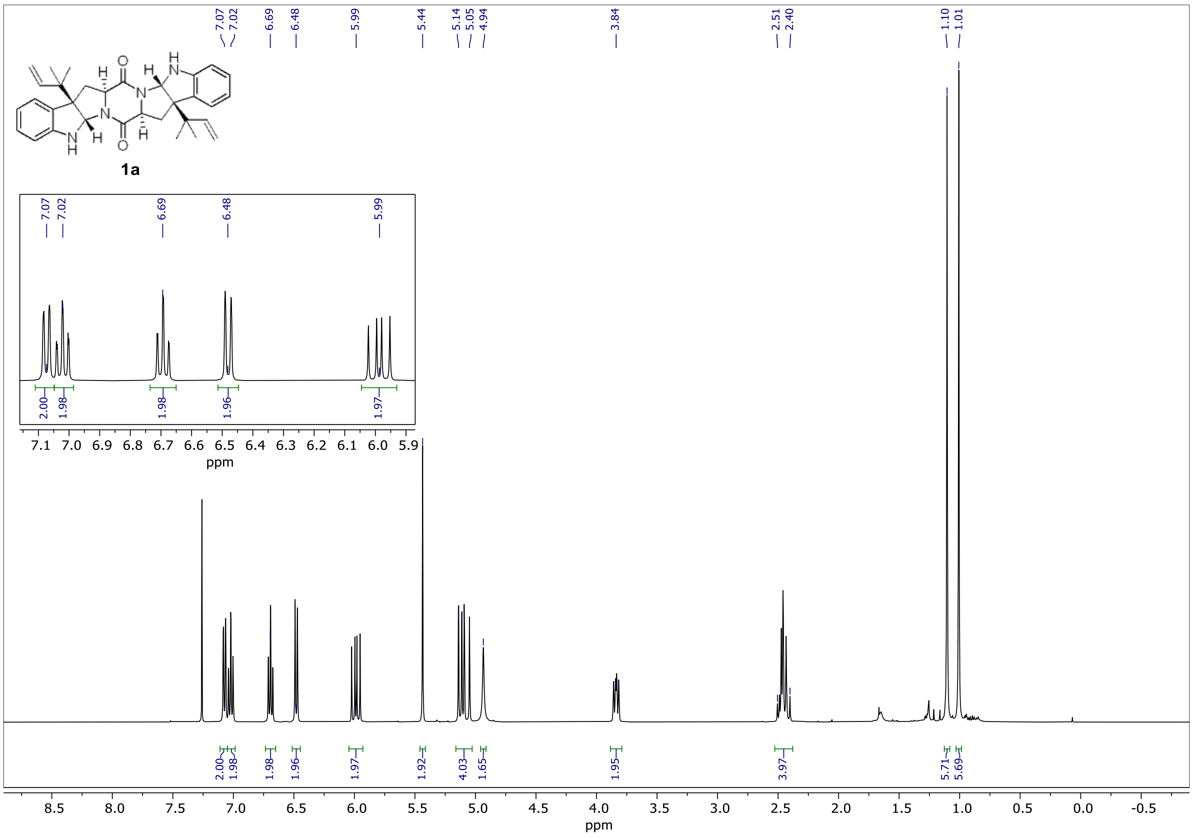

Spectrum 23. ^{1}H-NMR (400 MHz, $CDCl_3$) of **1a**.

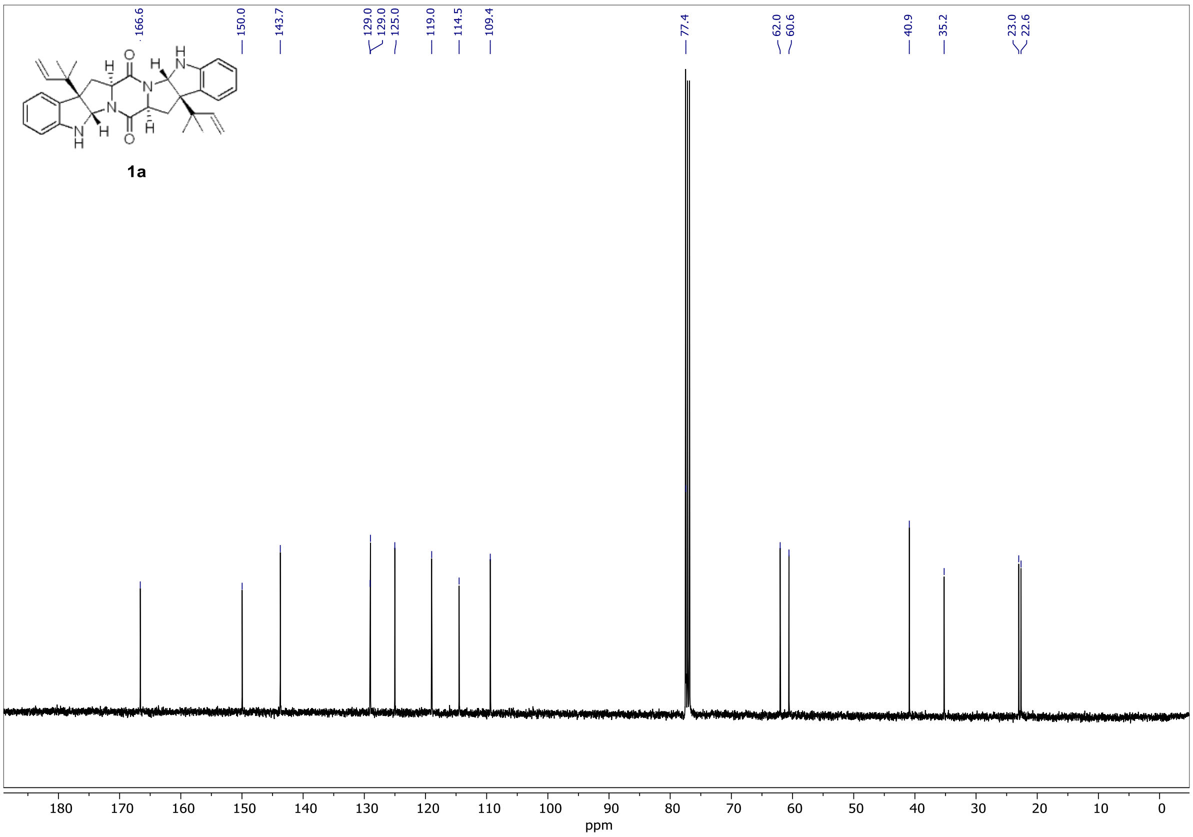

Spectrum 24. ^{13}C-NMR (101 MHz, $CDCl_3$) of **1a**.

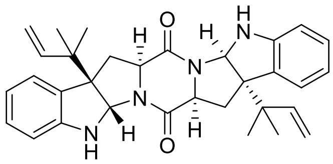

(-)-Epiamauromine (**1b**)

R*f*: 0.43 (PE/EtOAc = 3:1). **Mp.**: 154-155 °C. $[\alpha]_D^{20}$ = - 107.2 ° (*c* = 1.0, $CHCl_3$). **^{1}H-NMR** (400 MHz, $CDCl_3$): δ = 7.18 (dd, ^3J = 7.5 Hz, ^4J = 1.2 Hz, 1H), 7.13 (dd, ^3J = 7.5 Hz, ^4J = 1.2 Hz, 1H), 7.10 (dt, ^3J = 7.8 Hz, ^4J = 1.0 Hz, 1H), 7.08 (dt, ^3J = 7.8 Hz, ^4J = 1.0 Hz, 1H), 6.75 (dt, ^3J = 7.5 Hz, ^4J = 1.0 Hz, 1H), 6.74 (dt, ^3J = 7.5 Hz, ^4J = 1.0 Hz, 1H), 6.57 (d, ^3J = 7.8 Hz, 1H), 6.55 (d, ^3J = 7.8 Hz, 1H), 5.93 (dd, $^3J_{trans}$ = 17.2 Hz, $^3J_{cis}$ = 10.9 Hz, 1H), 5.89 (dd, $^3J_{trans}$ = 17.2 Hz, $^3J_{cis}$ = 10.9 Hz, 1H), 5.39 (s, 1H), 5.33 (s, 1H), 5.32 (s, 1H), 5.11 (dd, $^3J_{cis}$ = 10.8 Hz, ^5J = 0.9 Hz, 1H), 5.09 (dd, $^3J_{cis}$ = 10.8 Hz, ^5J = 1.0 Hz, 1H), 5.08 (dd, $^3J_{trans}$ = 17.3 Hz, ^5J = 0.9 Hz, 1H), 5.03 (dd, $^3J_{trans}$ = 17.3 Hz, ^5J = 1.0 Hz, 1H), 4.98 (s, 1H), 4.05 (dt, ^3J = 9.1 Hz, ^4J = 1.9 Hz, 1H), 3.91 (ddd, ^3J = 11.1 Hz, ^3J = 6.3 Hz, ^4J = 1.8 Hz, 1H), 2.77 (dd, ^2J = 13.9 Hz, ^3J = 8.7 Hz, 1H), 2.52 (dd, ^2J = 12.7 Hz, ^3J = 6.5 Hz, 1H), 2.46 (dd, ^2J = 14.0 Hz, ^3J = 9.5 Hz, 1H), 2.38 (dd, ^2J = 12.7 Hz, ^3J = 11.0 Hz, 1H), 1.12 (s, 3H), 1.07 (s, 3H), 0.97 (s, 3H), 0.95 (s, 3H) ppm. **^{13}C-NMR** (101 MHz, $CDCl_3$): δ = 168.7, 166.4, 150.0, 148.6, 143.6, 143.5, 131.7, 129.2, 129.0, 128.4, 125.8, 125.1, 118.9, 118.7, 114.8, 114.6, 109.4, 108.9, 79.4, 77.3, 62.2, 62.1, 60.8, 59.2, 41.8, 40.9, 36.1, 35.3, 23.0, 22.7, 22.6, 22.5 ppm. **FT-IR** (ATR): $\tilde{\nu}$ = 3339, 2972, 2929, 1744, 1655, 1458, 1413, 1387, 1369, 1249, 1147, 1010, 839, 741 cm^{-1}. **HR-MS** (ESI) *m/z*: $[M+H]^+$ calc. for $C_{32}H_{37}N_4O_2^+$ = 509.2911, found = 509.2918.

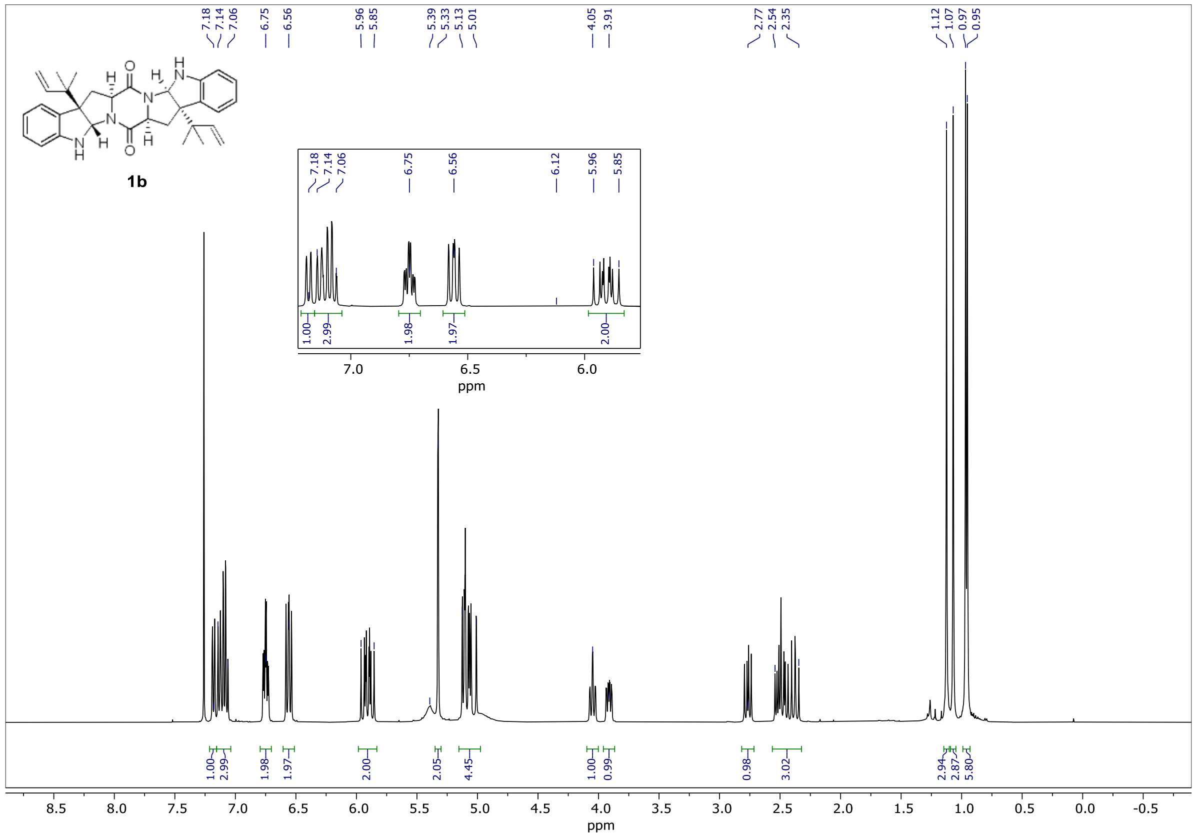

Spectrum 25. ^{1}H-NMR (400 MHz, $CDCl_3$) of **1b**.

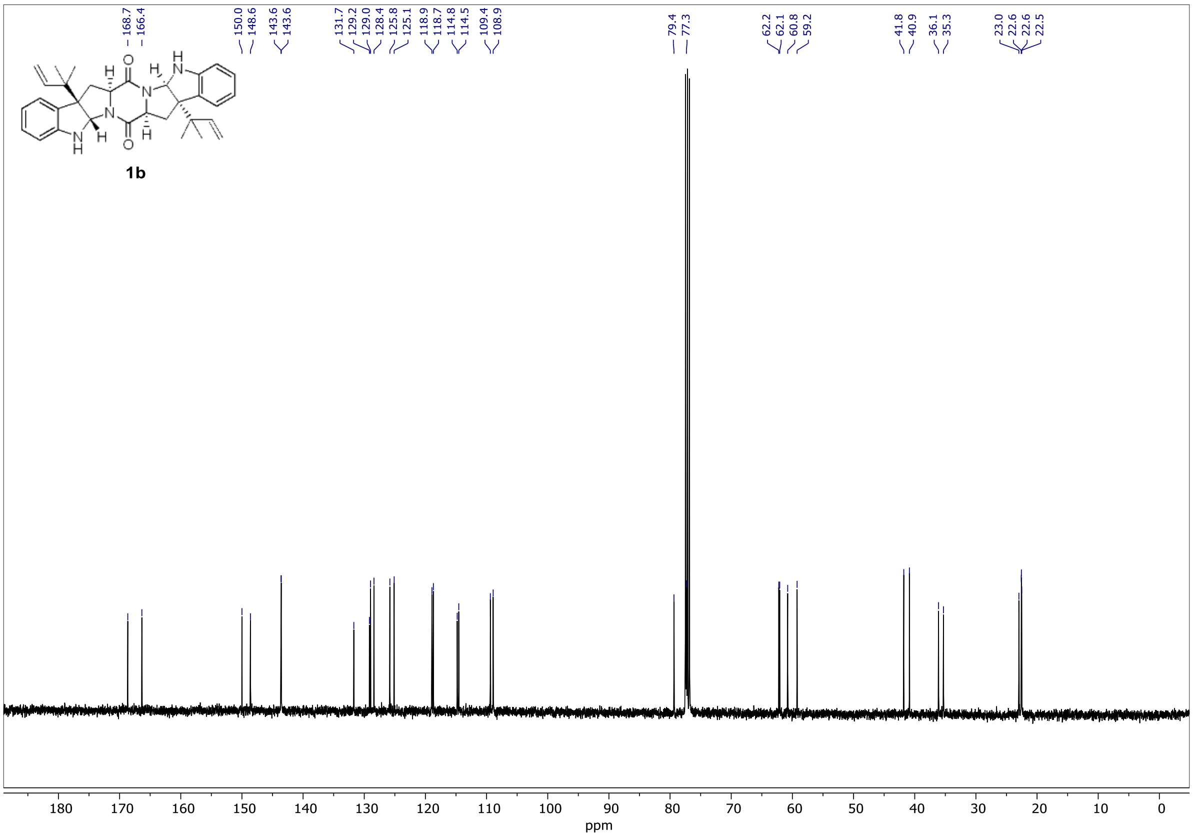

Spectrum 26. ^{13}C-NMR (101 MHz, $CDCl_3$) of **1b**.

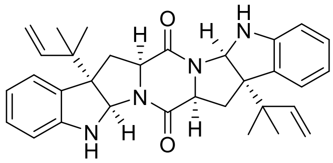

(+)-Novoamauromine (**1c**)

R$_f$: 0.26 (PE/EtOAc = 3:1). **Mp.**: 170-171 °C. $[\alpha]_D^{20}$ = + 285.9 ° (c = 1.0, $CHCl_3$). **^{1}H-NMR** (400 MHz, $CDCl_3$): δ = 7.12 (dd, ^3J = 7.5 Hz, ^4J = 1.2 Hz, 2H), 7.01 (dt, ^3J = 7.6 Hz, ^4J = 1.2 Hz, 2H), 6.70 (dt, ^3J = 7.5 Hz, ^4J = 1.1 Hz, 2H), 6.31 (dd, ^3J = 7.8 Hz, ^4J = 1.0 Hz, 2H), 5.92 (dd, $^3J_{trans}$ = 17.4 Hz, $^3J_{cis}$ = 10.9 Hz, 2H), 5.27 (s, 2H), 5.12 (dd, $^3J_{cis}$ = 11.0 Hz, ^5J = 1.0 Hz, 2H), 5.09 (s, 2H), 5.08 (dd, $^3J_{trans}$ = 17.5 Hz, ^5J = 1.0 Hz, 2H), 4.08 (dd, ^3J = 9.0 Hz, ^3J = 7.4 Hz, 2H), 2.71 (dd, ^2J = 13.8 Hz, ^3J = 9.3 Hz, 2H), 2.54 (dd, ^2J = 13.8 Hz, ^3J = 7.3 Hz, 2H), 1.10 (s, 6H), 0.93 (s, 6H) ppm. **^{13}C-NMR** (101 MHz, $CDCl_3$): δ = 168.3, 148.5, 143.9, 130.8, 128.5, 125.6, 118.6, 114.7, 109.6, 79.3, 62.5, 59.8, 41.7, 34.8, 22.7, 22.5 ppm. **FT-IR** (ATR): $\tilde{\nu}$ = 2923, 1752, 1657, 1444, 1387, 1371, 1252, 1148, 1007, 917, 839, 739 cm^{-1}. **HR-MS** (ESI) m/z: $[M+H]^+$ calc. for $C_{32}H_{37}N_4O_2^+$ = 509.2911, found = 509.2918.

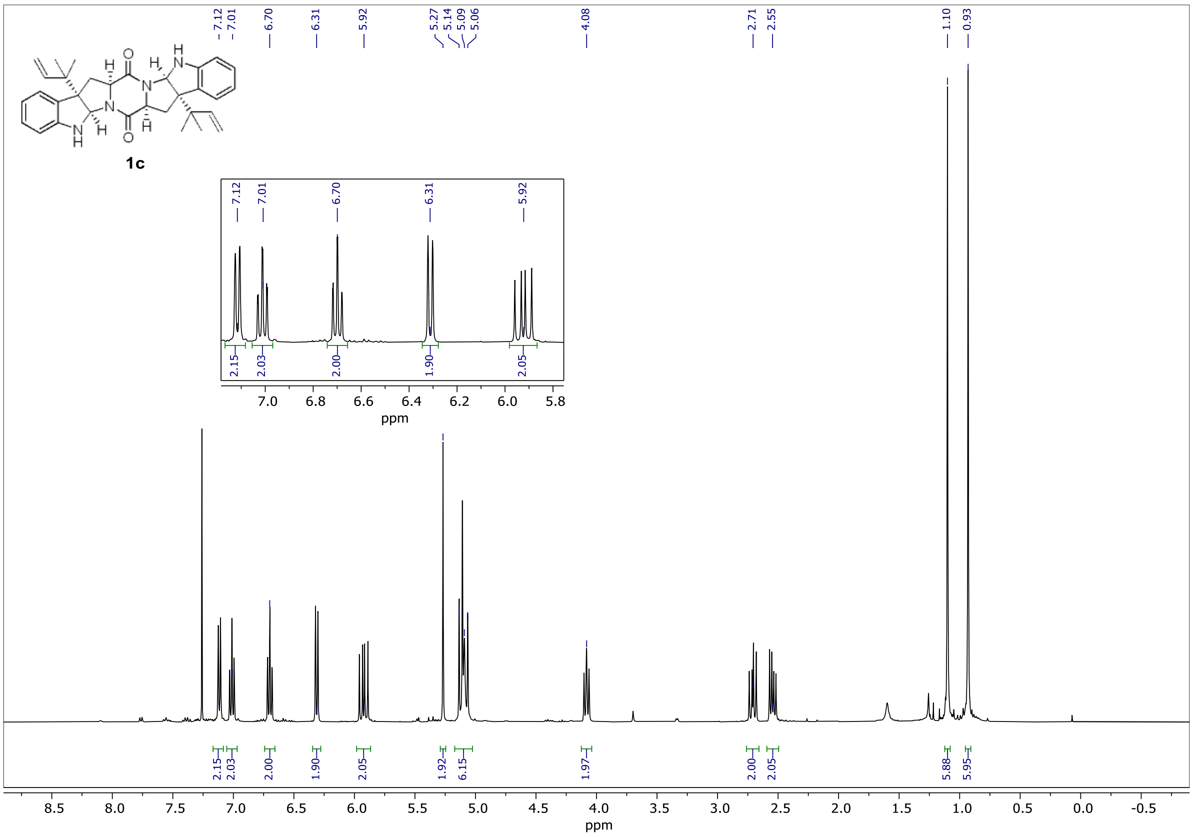

Spectrum 27. ^{1}H-NMR (400 MHz, $CDCl_3$) of **1c**.

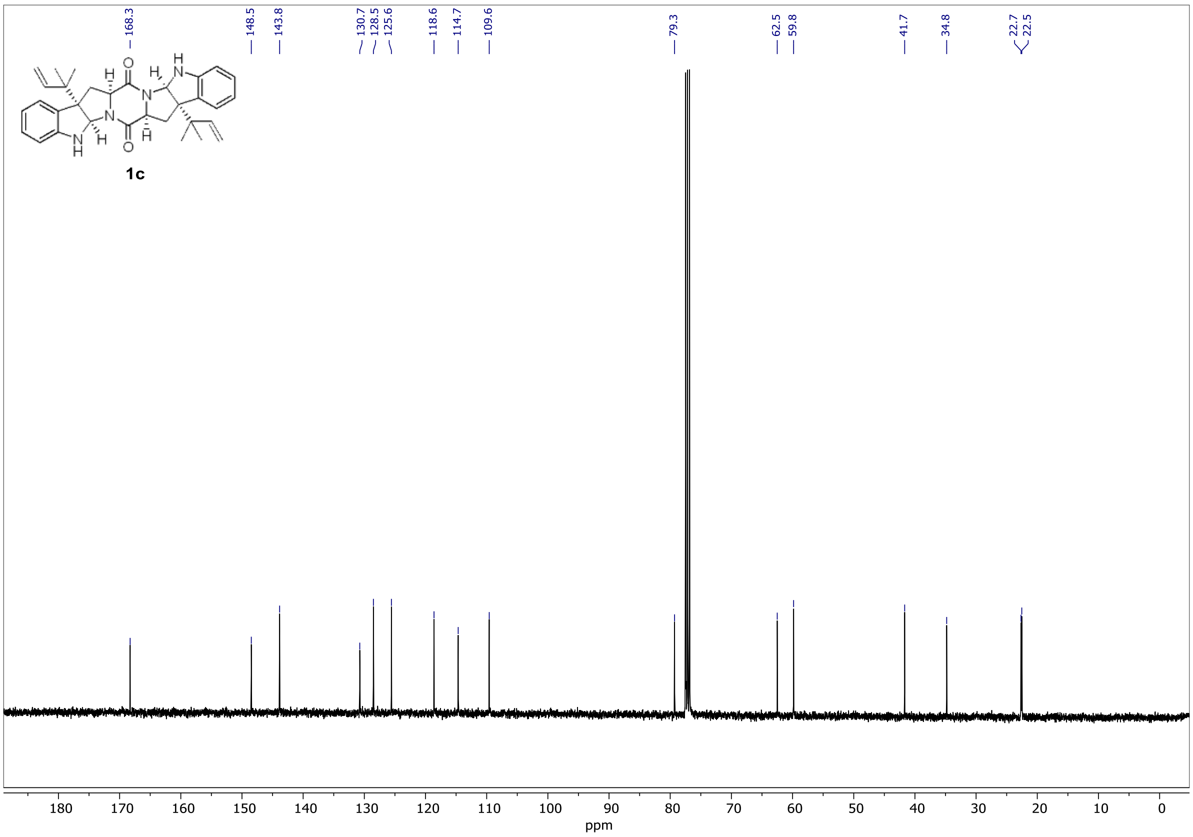

Spectrum 28. ^{13}C-NMR (101 MHz, $CDCl_3$) of **1c**.

3 References

[1] M. E. Kieffer, K. V. Chuang, S. E. Reisman, *J. Am. Chem. Soc.* **2013**, *135*, 5557–5560.

[2] F. Jubeen, S. Ijaz, I. Jabeen, U. Aftab, W. Mehdi, A. Altaf, S. A. Alissa, H. A. Al-Ghulikah, S. Ezzine, I. Bejaoui, M. Iqbal, *Arab. J. Chem.* **2022**, *15*, 104299.

[3] T. Iizuka, S. Takiguchi, Y. Kumakura, N. Tsukioka, K. Higuchi, T. Kawasaki, *Tetrahedron Lett.* **2010**, *51*, 6003–6005.
